# Force-induced charge carrier storage: a new route for stress recording

**DOI:** 10.1038/s41377-020-00422-4

**Published:** 2020-10-27

**Authors:** Yixi Zhuang, Dong Tu, Changjian Chen, Le Wang, Hongwu Zhang, Hao Xue, Conghui Yuan, Guorong Chen, Caofeng Pan, Lizong Dai, Rong-Jun Xie

**Affiliations:** 1 College of Materials, Xiamen University, Simingnan-Road 422, Xiamen, 361005 China; 2 Fujian Provincial Key Laboratory of Materials Genome, Xiamen University, Simingnan-Road 422, Xiamen, 361005 China; 3 School of Physics and Technology, Wuhan University, Bayi-Road 299, Wuhan, 430072 China; 4 College of Optical and Electronic Technology, China Jiliang University, Xueyuan-Street 258, Hangzhou, 310018 China; 5 Institute of Urban Environment, Chinese Academy of Sciences, Jimei-Avenue 1799, Xiamen, 361021 China; 6 Fujian Provincial Key Laboratory of Fire Retardant Materials, Xiamen University, Simingnan-Road 422, Xiamen, 361005 China; 7 Beijing Institute of Nanoenergy and Nanosystems, Chinese Academy of Sciences, Xueyuan-Road 30, Beijing, 100083 China

**Keywords:** Imaging and sensing, Optical data storage, Optical sensors, Inorganic LEDs

## Abstract

Stress sensing is the basis of human-machine interface, biomedical engineering, and mechanical structure detection systems. Stress sensing based on mechanoluminescence (ML) shows significant advantages of distributed detection and remote response to mechanical stimuli and is thus expected to be a key technology of next-generation tactile sensors and stress recorders. However, the instantaneous photon emission in ML materials generally requires real-time recording with a photodetector, thus limiting their application fields to real-time stress sensing. In this paper, we report a force-induced charge carrier storage (FICS) effect in deep-trap ML materials, which enables storage of the applied mechanical energy in deep traps and then release of the stored energy as photon emission under thermal stimulation. The FICS effect was confirmed in five ML materials with piezoelectric structures, efficient emission centres and deep trap distributions, and its mechanism was investigated through detailed spectroscopic characterizations. Furthermore, we demonstrated three applications of the FICS effect in electronic signature recording, falling point monitoring and vehicle collision recording, which exhibited outstanding advantages of distributed recording, long-term storage, and no need for a continuous power supply. The FICS effect reported in this paper provides not only a breakthrough for ML materials in the field of stress recording but also a new idea for developing mechanical energy storage and conversion systems.

## Lung disease: Mechanoluminescence: stress-sensitive phosphors hold deep memories

A material that records mechanical impacts and provides optical readouts at later dates shows promise for anti-counterfeiting devices and structural damage analysis. Phosphors, such as rare earth-doped silicates, can emit light in response to physical stress because they store charge carriers in easy-to-access energy states. Rong-Jun Xie from China’s Xiamen University and colleagues now report development of phosphors that release charges to less accessible ‘deep’ energy states after being stimulated mechanically. These carriers are retained in the deep states and then released on-demand as photon emissions following thermal treatments. The team demonstrated several applications for the new phosphors including sensors that can record signature traces and composite films that attach to vehicles to monitor for potential collisions.

## Introduction

Mechanoluminescence (ML) materials featuring photon emission under mechanical stimuli have attracted the attention of material scientists, physicists and engineers^1–6^ due to their promising applications in structure damage diagnosis platforms, E-signature systems, artificial skins and optical anti-counterfeiting devices^7–12^. By using photons as mediators, ML materials could enable remote stress sensing (no contact between the pressed units and detectors)^13,14^ as well as distributed monitoring for large-scale surfaces or complex interfaces^15,16^, which are still great challenges for conventional stress sensing methods based on the piezoelectric effect and resistance or capacitance changes. Generally, ML-based stress sensing is a real-time sensing method, giving a fast response to an applied mechanical stimulus^17,18^. Non-real-time stress sensing (also known as stress recording) is also urgently required in many important applications, especially when mechanical action occurs at an unexpected moment (e.g., collision recording in vehicle accidents or falling point monitoring in ball games). These applications, however, cannot be achieved with existing real-time ML stress sensing systems.

**Fig. 1:**
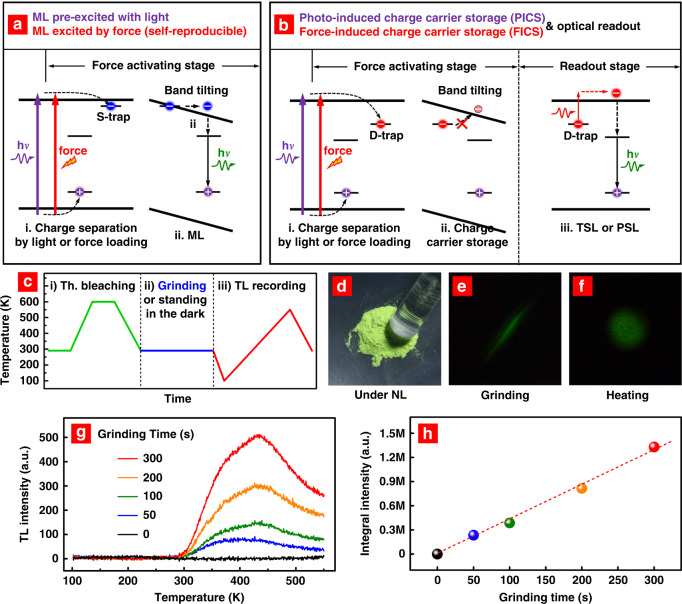
Phenomenon and mechanism of the FICS effect. **a** Energy-level diagram for the ML mechanism. The charge carriers are excited to shallow traps by light pre-irradiation (purple arrow) or by force loading (red arrow). ML is generated under the piezoelectricity-induced energy band tilting. **b** Energy-level diagram showing the FICS effect, PICS effect, and optical read-out in deep-trap ML materials. The charge carriers are excited to deep traps either by light pre-irradiation (purple arrow) or force loading (red arrow) for the PICS and FICS processes, respectively. The deep-trapped charge carriers are not released even under band tilting. **c** Temperature curve for the demonstration experiments of the FICS effect. The samples were pre-heated to 600 K to remove charge carriers from traps before grinding. **d**–**f** Photographic images of the SrSi_2_O_2_N_2_:Eu^2+^,Dy^3+^ phosphor under natural light (NL), grinding (after thermal bleaching) and heating (after grinding). **g** TL glow curves after grinding the phosphor for different times. **h** Integral TL intensity as a function of the grinding time

ML is generally accepted to be closely related to the interactions between charge carriers and shallow traps (Fig. 1a)^19–21^. Typically, charge carriers are pumped into shallow traps by light pre-excitation^22–25^ or force loading^26–30^ (Fig. 1a:i). The charge carriers are released from shallow traps at an accelerated rate under force loading, leading to instantaneous photon emission. The accelerated release of charge carriers is related to trap depth reduction. In this regard, an energy model of band titling has been widely used to explain the trap depth reduction, in which the band configuration varies under a piezoelectricity-induced electric field (Fig. 1a:ii)^31–33^. On the other hand, deep traps in ML materials have not yet been fully exploited. Recently, Smet et al. reported a ground-breaking approach to record mechanical action by using deep traps in ML materials, which exhibits great potential in stress recording^34^. In this work, the ML materials were pre-excited by ultraviolet (UV) light and stressed on the surface by dragging a rod. The applied stress was recorded in the materials because it re-distributed the charge carriers from shallow to deep traps.

Our group has introduced deep traps into (Sr,Ba)Si_2_O_2_N_2_ through trap depth engineering and demonstrated applications in optical information storage by using these deep traps to capture and store charge carriers^35,36^. Meanwhile, several studies have revealed that the compound (Sr,Ba)Si_2_O_2_N_2_ is also an excellent host for ML materials^34,37^. This work inspired us to combine deep-trap materials with ML materials and raised the question of whether the deep traps in ML materials can capture and store charge carriers excited by mechanical stimuli. The answer to this question may lead us to another solution for achieving stress recording without pre-excitation of light.

Herein, for the first time, we report a force-induced charge carrier storage effect (hereinafter denoted the FICS effect) in deep-trap ML materials (Fig. 1b). The FICS occurs when the charge carriers of luminescent centres are excited by a force, captured in deep traps, and stored in the materials at room temperature (RT). The stored charge carriers can be released under high-temperature thermal stimulation, producing emission. The FICS effect enables the ML materials to store energy upon application of a mechanical stimulus and convert it to photons when needed, therefore enabling development of novel stress recording devices without a continuous electronic power supply. This work not only solves the shortcomings of ML materials in stress recording applications but also opens up a new path for designing multifunctional materials with energy storage and energy conversion capabilities.

## Results

### FICS effect in SrSi_2_O_2_N_2_:Eu^2+^,Dy^3+^

In a typical measurement procedure, phosphor samples were heat-treated at 600 K (see Fig. 1c:i), ground in the dark by using an agate rod at RT (ii), and heated again for thermoluminescence (TL) tests (iii). The first heating step was indispensable because all the charge carriers were expected to be removed from traps before the mechanical stimulus was applied. This step was called thermal bleaching. We started with the green-light phosphor SrSi_2_O_2_N_2_:Eu^2+^,Dy^3+^ (Fig. 1d). When the thermally bleached phosphors were gently ground in the dark, green ML could be clearly observed by the naked eye (Fig. 1e). The green ML disappeared once the grinding stopped. When the ground phosphors were moved to a heating stage at 200 °C, amazingly, they exhibited green emission (Fig. 1f). We measured the TL glow curves after grinding for different grinding times. As depicted in Fig. 1g, no TL signal was observed without grinding, whereas a broad TL band covering 300–550 K was detected after grinding. The integral TL intensity increased with grinding time, showing a linear relationship (Fig. 1h). Based on the above experimental results, we preliminarily proved that the FICS effect exists in the SrSi_2_O_2_N_2_:Eu^2+^,Dy^3+^ phosphor, i.e., the charge carriers can be excited by a mechanical stimulus and then captured and stored in the deep traps of the ML materials.

### Charge carrier transitions in SrSi_2_O_2_N_2_:Eu^2+^,Dy^3+^ under force loading

**Fig. 2:**
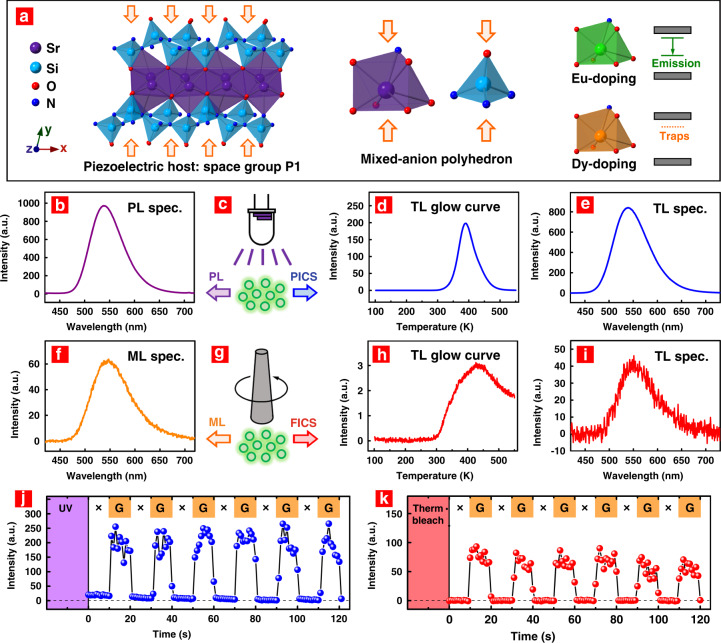
Charge carrier transitions in the deep-trap ML material SrSi_2_O_2_N_2_:Eu^2+^,Dy^3+^ under light irradiation or force loading. **a** Crystal structure of SrSi_2_O_2_N_2_:Eu^2+^,Dy^3+^. The Eu^2+^ and Dy^3+^ ions were incorporated into the Sr^2+^ sites as emitting centres and deep traps, respectively. **b** PL spectra under UV irradiation. **c** Schematic diagram for PL and PICS. **d**, **e** TL glow curve and TL spectra recorded after UV irradiation. **f** ML spectra under force loading. **g** Schematic diagram for ML and FICS. **h**, **i** TL glow curve and TL spectra recorded after force loading. **j**, **k** Emission intensity in response to force loading (grinding). The symbols “×” and “G” along the time axis represent no grinding and grinding, respectively. The samples were pre-excited by UV light in (**j**), while they were thermally bleached before the test in (**k**)

According to the XRD characterization (Fig. S1), the SrSi_2_O_2_N_2_ crystal is triclinic with the space group of P1 and is composed of [SrO_6_N] and [SiON_3_] mixed-anion polyhedrons connected in a layered structure (Fig. 2a)^38^. The mixed-anion polyhedrons offer local asymmetry for the central cations and form an asymmetric piezoelectric crystal structure by periodic arrangement^39^. Regarding the emitting centre, Eu^2+^ showed large absorbance in the wavelength range from 250 to 450 nm and gave high-efficiency green emission in SrSi_2_O_2_N_2_ (Fig. S2)^40,41^. Dy^3+^ was codoped in the host to create deep traps^35,36^.

To further verify the FICS effect in SrSi_2_O_2_N_2_:Eu^2+^,Dy^3+^, we compared the effects of light irradiation and force loading on the charge carrier transitions (Fig. 2b–i). The PL spectrum under UV irradiation (Fig. 2b) and the ML spectrum under grinding (Fig. 2f) showed the same green emission peak at ~ 540 nm. The TL glow curve after UV irradiation exhibited a glow band covering 300 to 500 K (Fig. 2d). The TL band after grinding at RT was located in a similar temperature range, but it was slightly broadened and shifted to a higher temperature (Fig. 2h). The TL spectra after UV irradiation and mechanical grinding (Fig. 2e, i) were almost identical to the PL and ML spectra, confirming that Eu^2+^ was the recombination (emission) centre for both the light-induced and force-induced excitations. The mechanism of photo-induced charge carrier storage (PICS) has been investigated in many materials, with the conclusion that deep traps can store light-excited charge carriers and exhibit one or more TL glow peaks when temperatures above RT are reached^42–44^. The similar TL glow curves (Fig. 2d, h) clearly indicated that those deep traps responsible for the PICS could also store the charge carriers excited by force. Although force and light were both accessible excitation sources for charge carrier storage, the excitation efficiency induced by the force loading in SrSi_2_O_2_N_2_:Eu^2+^,Dy^3+^ was much lower than that induced by light irradiation. Exploring more materials with a higher excitation efficiency for the FICS effect is thus necessary.

As depicted in Fig. 2j, k, regardless of whether it was pre-irradiated by UV light, SrSi_2_O_2_N_2_:Eu^2+^,Dy^3+^ showed ML under force loading. However, the ML intensity after UV pre-irradiation (Fig. 2j) was higher than that after thermal bleaching (Fig. 2k). The higher intensity should be ascribed to the accelerated release under force loading of stored charge carriers that had been pumped to deep traps under the UV pre-irradiation. Therefore, the applied stress may drive the charge carrier transitions in two directions. One direction involves excitation of the charge carriers and storage of some of them in deep traps. The other direction involves acceleration of the release of charge carriers, especially from shallower traps. These two effects slightly shifted the TL glow peak recorded after force loading towards higher temperature compared with that after UV irradiation (see Fig. 2d, h). It should be noted that the average ML intensity in Fig. 2k basically remained unchanged but appeared to slightly decrease with the grinding time. The slight decrease in the ML intensity should be due to slight damage to particles during the grinding.

### FICS effect in other materials

**Fig. 3:**
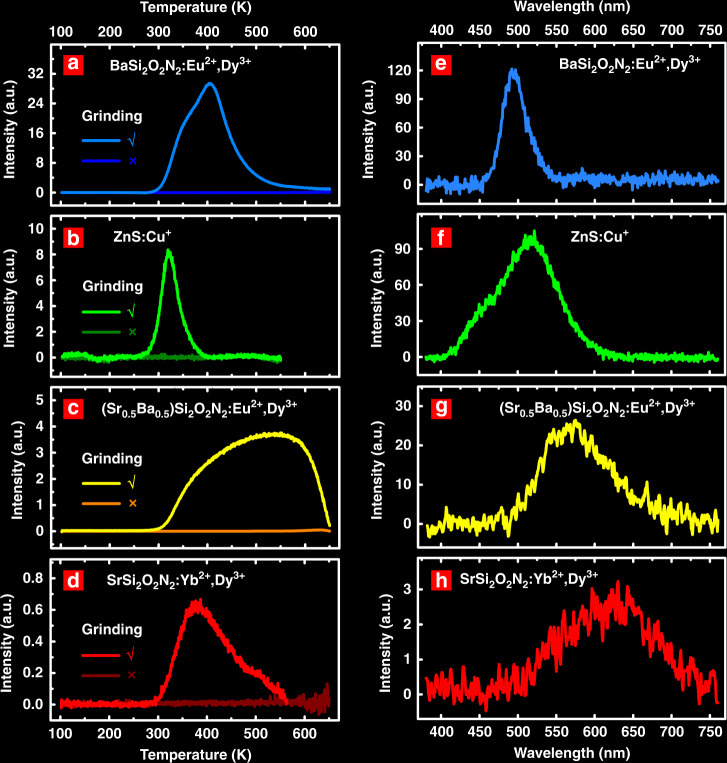
FICS effect in various materials. **a**–**d** TL glow curves after grinding the phosphors for 200 s (√) and without grinding (×). Before the TL measurements, all the samples were thermally bleached at 700 K. **e**–**h** Force-induced TL spectra. The emission colour could be tailored from blue to green, yellow and red by using various materials

Furthermore, we studied the FICS effect in the phosphors BaSi_2_O_2_N_2_:Eu^2+^,Dy^3+^, ZnS:Cu, (Sr_0.5_Ba_0.5_)Si_2_O_2_N_2_:Eu^2+^,Dy^3+^, and SrSi_2_O_2_N_2_:Yb^2+^,Dy^3+^. As shown in Fig. 3a–d, they showed high-temperature TL bands after grinding at RT. The force-induced TL spectra (Fig. 3e–h) were similar to the PL and ML spectra (Figs. S3, S4). The spectra could be tailored from blue (BaSi_2_O_2_N_2_:Eu^2+^,Dy^3+^) to green (ZnS:Cu), yellow ((Sr_0.5_Ba_0.5_)Si_2_O_2_N_2_:Eu^2+^,Dy^3+^) and red (SrSi_2_O_2_N_2_:Yb^2+^,Dy^3+^) light according to the species of the emission centres and the surrounding crystal fields. All the phosphors possessed piezoelectric crystal structures (BaSi_2_O_2_N_2_: orthorhombic Pbcn^45^, wurtzite-ZnS: hexagonal P6mm^46^ and SrSi_2_O_2_N_2_: triclinic P1^38^), efficient luminescent centres (Eu^2+^, Cu^+^, and Yb^2+^), and deep traps. Although shallow traps might coexist in these materials (such as in BaSi_2_O_2_N_2_:Eu^2+^,Dy^3+^ and ZnS:Cu; see Figs. S5, S6), the charge carriers could be stored only in the relatively deep traps under force loading at RT.

The force-induced TL glow curves of the studied materials indicated that BaSi_2_O_2_N_2_:Eu^2+^,Dy^3+^ and ZnS:Cu showed higher TL intensities than the other materials (Fig. S7). This result was consistent with the fact that these two phosphors presented stronger ML without UV pre-irradiation (Fig. S8). Thus, the excitation efficiency of charge carriers under force loading should be an important factor for the FICS effect.

### Applications of the FICS effect in stress recording

**Fig. 4:**
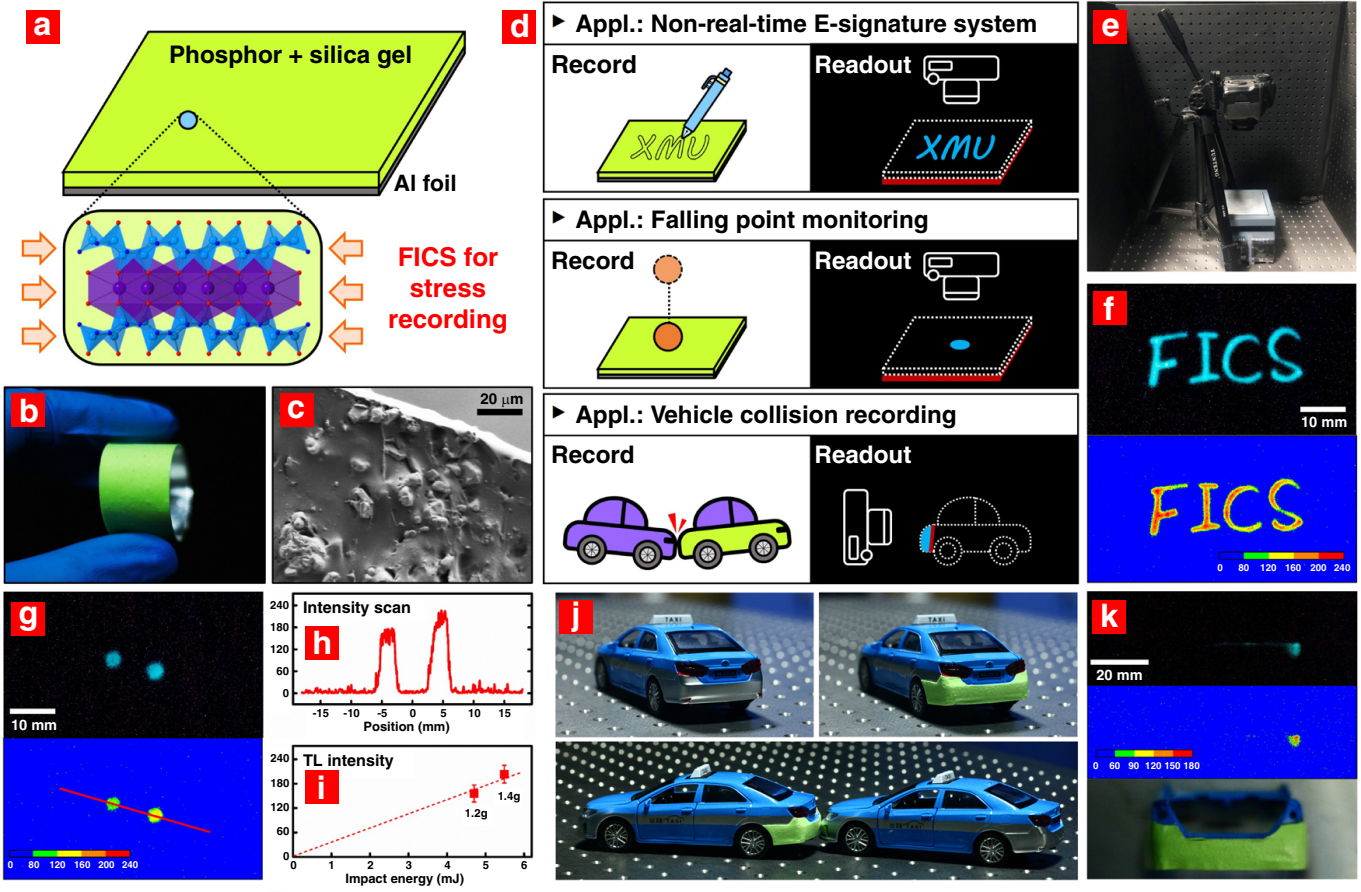
Applications of the FICS effect in stress recording. **a** Schematic diagram of a composite film containing the deep-trap ML phosphor BaSi_2_O_2_N_2_:Eu^2+^,Dy^3+^ and silica gel. The composite film was coated on a thin layer of aluminium foil for better heat conduction. The inset shows the crystal structure of BaSi_2_O_2_N_2_:Eu^2+^,Dy^3+^. **b**, **c** Photographic and SEM images of the composite film. **d** Schematic diagrams for three stress recording applications. The composite film was able to record various mechanical actions and store energy at RT. Then, the force-induced TL image was read out by a digital camera when the film was heated to 200 °C. **e** Reading device. **f**, **g** TL images and TL intensity maps. The film had “F I C S” written on it by using an ink-free pen in (**f**). The average force applied on the film was estimated to be ~0.2 N. The film was hit by two falling balls in (**g**). The mechanical (impact) energy applied on the film was 4.7 and 5.5 mJ. **h** Relative intensity along the straight line marked in (**g**). **i** TL intensity versus weight of the balls. **j** Model car before/after coating the composite film (top), and two cars in a rear-end collision accident (bottom). The impact energy produced by the collision was ~100 mJ. **k** TL image (top) and TL intensity map (middle) of the rear after a collision accident. The bottom is the same view field under natural light

We adopted BaSi_2_O_2_N_2_:Eu^2+^,Dy^3+^ to demonstrate the FICS effect in stress recording applications. A thin composite film of BaSi_2_O_2_N_2_:Eu^2+^,Dy^3+^@silica gel was prepared on a layer of aluminium foil by using a blade-coating method (Fig. 4a and ‘Materials and methods’ section for more details). This composite film could be maintained at 300 °C for several hours without perceived deterioration^35^. In addition, the composite film exhibited excellent flexibility and machinability (Fig. 4b, c) and thus could be utilized in a variety of fields.

Here, we show three examples of stress recording applications, as schematically depicted in Fig. 4d. Basically, the composite film with the FICS effect was used as a memory medium to record mechanical actions. The force-induced TL image was then read out by a digital camera when heating the composite film (typically 200 °C) to release the stored charge carriers from deep traps (the reading device is shown in Fig. 4e). In the first example, the composite film was signed by using an ink-free pen (e.g., writing the four letters “F I C S”) with an average loading force of ~0.2 N. Then, the film was kept at RT for at least 10 min. When it was placed on a heating stage, the entire signature track was clearly read out in the TL imaging (Fig. 4f). The force distribution could be obtained by mapping the TL intensity (Fig. 1h) since the TL intensity showed a linear relationship with the applied mechanical energy. Meanwhile, the previous signature trace could be removed by heating the film to a higher temperature (300 °C for BaSi_2_O_2_N_2_:Eu^2+^,Dy^3+^), and the film was recyclable for recording new signatures (see the three more signatures read out from the same film in Figs. S9–S12).

In the second example, the composite film was used to monitor falling points. Two balls with different weights (1.20 and 1.40 g, Fig. S13) were freely dropped from 40 cm above the film. Two circular spots were read out in the TL image (Fig. 4g), which were recorded on the film when the balls hit it. The TL intensity map in Fig. 4h indicates that different energies were stored for the two free-fall impacts. Notably, the TL intensity around the spot centre was nearly proportional to the impact energy received from the two falling balls (4.7 and 5.5 mJ, Fig. 4i). Therefore, not only the location but also the impact energy of a falling object could be estimated by constructing a relationship between the TL intensity and the applied mechanical energy.

Finally, we took two model cars to simulate vehicle collision accidents. A composite film was carefully coated on the rear of one car (Fig. 4j). In the dark, the film-coated car was rear-ended by the other car with an impact energy of ~100 mJ (weight 0.20 kg, velocity ~1 m/s). Then, the rear was removed and placed on a heating stage to read out optical signals. As shown in Fig. 4k, the TL image and TL intensity map, corresponding to the collision position and force distribution, were clearly read. Similar to the first example, the recording and read-out of a signal could be repeated after thermal regeneration as long as the composite film was not damaged. The TL imaging of two more collisions for the same car is given in Figs. S14–S16.

## Discussion

In this work, we demonstrated stress recording in five thermally bleached ML materials. The FICS effect was proposed to explain the stress recording phenomenon, which is schematically depicted in the energy-level diagram of Fig. 1b. Specifically, some charge carriers are excited to the conduction band under a mechanical action (red arrow), part of which are captured by deep traps. These charge carriers remain in the deep traps after force loading and are stored in the materials at RT. Finally, they can be quickly released at an elevated temperature as photon emission. The proposed FICS is similar to the optical storage process (purple arrow) in deep-trap persistent luminescence materials as published in ref. ^35^, in which the same materials except for ZnS:Cu were used for the study. However, a question is raised regarding how the mechanical actions can excite the charge carriers over the bandgap with a typical value of 4–6 eV.

Based on the results of this work, we consider that triboelectricity is possibly the main reason for the force-induced excitation. First, the five phosphors exhibited ML after thermal bleaching at 600 K (the phosphors were ground in the dark at RT; see Fig. 1e and Fig. S8). Among them, ZnS:Cu showed the highest intensity; the ML in SrSi_2_O_2_N_2_:Yb^2+^,Dy^3+^ was the weakest but still detectable. Recently, Sohn et al. investigated the origin of the self-producible ML (i.e., ML without pre-excitation of light) in a ZnS:Cu@PDMS (polydimethylsiloxane) composite and proposed the mechanism of triboelectricity-induced luminescence. According to that work, triboelectricity was generated during the frictional contact between the ZnS:Cu particles and the PDMS matrix. The friction-induced electric field further excited the phosphors and produced emission. The triboelectricity-induced luminescence answered the questions of why light pre-excitation was not necessary and why a small force load (such as 5N) was sufficient to produce ML^47^. Since the charge carriers had been emptied from traps (at least all the traps with TL temperatures below 600 K), the observed ML without light pre-excitation in Fig. 1e and Fig. S8 could also be attributed to triboelectricity. Importantly, the generation of triboelectricity requires fast friction between two solids. When grinding the phosphors, the phosphor particles were violently rubbed against the agate and other particles. For the impact and load dragging on the composites, there was also fast friction between the particle surfaces and soft silica-gel matrix. Thus, the requirements of triboelectricity were well met in the demonstration of the FICS process. In contrast, when we slowly applied a compressive force of 0.2N (0.255 MPa) on the surface of a BaSi_2_O_2_N_2_:Eu^2+^,Dy^3+^ composite and maintained the static force for 60 s without dragging, we could not detect the FICS process as expected. This result indicated that a small static force was unable to excite the charge carriers over the bandgap. On the other hand, when fast friction was produced on the interface, a friction-induced electric field could be established to excite the charge carriers. According to Sohn et al., the electric field around the force-applied ZnS:Cu particle was roughly estimated to be 10^6^–10^7^ V/cm^47^, which is much higher than the threshold value required to excite electrons. However, accurate calculation of the resultant electric field in different materials is still very challenging, especially considering the dynamic characteristics and the force distribution.

In addition to the mechanism of triboelectricity-induced excitation, there are several other possible interpretations of the detected TL band after force loading. The first one is that the charge carriers may come from deeper traps. In this case, some charge carriers may have been captured by deeper traps, possibly due to unintentional light excitation, and they could not be removed by the thermal bleaching process (for example, at 600 K in Fig. 1c–h); upon force loading, they may be shifted to relatively shallow traps corresponding to the 300–600 K region in TL curves. We performed another TL measurement in SrSi_2_O_2_N_2_:Eu^2+^,Dy^3+^ after UV irradiation over a broader range from 300 to 800 K. We still could not find any TL signal higher than 600 K (Fig. S17). This result suggested that there are no deeper traps corresponding to the 600–800 K region. In addition, we pre-heated the SrSi_2_O_2_N_2_:Eu^2+^,Dy^3+^ phosphors at 800 K, ground them at RT, and obtained a TL glow curve with a broad band at ~400 K (Fig. S18), similar to Fig. 1g. Thus, we can conclude that the force-induced TL band at least does not come from charge carrier redistribution from traps with a depth less than ~2.5 eV (~800 K). Another possibility is that force charging occurs when the ML particles are fractured. Indeed, grinding of phosphors may lead to the fracture of some particles. On the other hand, when the phosphors were incorporated into silica gels, the particles were not damaged under force loading due to the buffering of the soft polymer matrix (see the SEM image after load dragging in Fig. S19). Meanwhile, the FICS and optical read-out were repeatable, as demonstrated in Fig. S12 (load dragging) and Fig. S16 (vehicle collision), which indicated that the force-induced storage in the composite was not a destructive process. Finally, the force charging could also originate from piezoelectricity (i.e., piezoelectricity-induced electroluminescence)^7,48^, especially considering the piezoelectric crystal structures of the studied ML materials. However, it is difficult to understand how the charge carriers could be excited by a dynamic force with significant movement but not by a static force (0.2 N). At present, we believe that the ML and FICS processes in this work are likely due to triboelectricity-induced excitation. Nevertheless, it is still difficult to exclude other possibilities. Many issues remain to be further studied in depth.

From the viewpoint of potential applications, the FICS effect proposed in this paper allows for distributed stress sensing in a non-real-time mode, therefore opening up a new route inaccessible for conventional stress sensing methods. Meanwhile, the FICS effect may create new opportunities for developing next-generation battery-free stress recording devices as well as mechanical energy storage and conversion systems.

In conclusion, we investigated the charge carrier transitions in SrSi_2_O_2_N_2_:Eu^2+^,Dy^3+^, BaSi_2_O_2_N_2_:Eu^2+^,Dy^3+^, ZnS:Cu, (Sr_0.5_Ba_0.5_)Si_2_O_2_N_2_:Eu^2+^,Dy^3+^ and SrSi_2_O_2_N_2_:Yb^2+^,Dy^3+^ and reported a new effect called force-induced charge carrier storage (FICS) in the five deep-trap ML materials. The FICS effect enabled the conversion of part of the applied mechanical energy into deep-trap-related states, which could be stored at RT and released as photon emission at higher temperatures. Detailed spectroscopic studies indicated that the charge carrier transition of the FICS effect was similar to the charge carrier storage in deep-trap persistent luminescence materials under light irradiation, although the FICS effect also required the materials to have significant ML characteristics. The analysis of the mechanism suggested that the FICS effect likely originated from the excitation of a triboelectricity-induced electric field. Importantly, the reported FICS effect has promise in non-real-time stress sensing applications, such as electronic signature recording, falling point monitoring and vehicle collision recording. The stress recording based on the FICS effect avoids the requirement of a continuous power supply and simplifies the system construction. Finally, the FICS effect provides a possible method of mechanical energy conversion and storage and is expected to be applied in the field of energy collection and utilization.

## Materials and methods

### Synthesis of deep-trap ML materials

The SrSi_2_O_2_N_2_:Eu^2+^,Dy^3+^, BaSi_2_O_2_N_2_:Eu^2+^,Dy^3+^, (Sr_0.5_Ba_0.5_)Si_2_O_2_N_2_:Eu^2+^,Dy^3+^ and SrSi_2_O_2_N_2_:Yb^2+^,Dy^3+^ phosphors were synthesized by using the two-step high-temperature solid-state reaction method reported in our previous work^35^. The sintering temperatures for the first and second steps were 1300 and 1550 °C, respectively. The doping concentrations were 2 mol% for Eu^2+^/Yb^2+^ and 1 mol% for Dy^3+^ in the four samples. The phosphor ZnS:Cu was obtained from Shanghai Keyan Phosphor Technology without further treatment.

### Preparation of composite films

The synthesized phosphors were ground and sieved (200 meshes/inch) to remove large particles. The phosphors were mixed with silica-gel precursors in a weight ratio of 1:1. The phosphor slurry was poured onto a thin aluminium foil and flattened to a smooth film using a scraper. The phosphor@silica-gel composite films were firmed in an oven at 80 °C for 1 h and 200 °C for 3 h. The thickness of the composite films was set to 1–2 mm. The prepared composite films showed excellent flexibility and machinability. They can be conveniently attached to the surfaces of metal or glass plates.

### Structural and optical characterizations

Powder XRD patterns of the phosphors were obtained by an X-ray diffractometer (Bruker, D8 Advance) with Cu K_α_ as the radiation source (*λ* = 0.15406 nm). The surface morphology of the composite film was characterized by field-emission scanning electronic microscopy (Hitachi, SU70). PL, ML and TL spectra were collected by a charge-coupled device (CCD) fibre spectrophotometer (Ocean Optics, QE Pro). UV-light-induced or force-induced TL glow curves were recorded via a homemade measurement system driven by a LabVIEW-based programme. A light-filter-attached photomultiplier tube (PMT) detector (Hamamatsu Photonics, R928), a cooling/heating stage (Linkam Scientific Instruments, THMS600E) and a Hg lamp (18 W) as the light source were integrated into the measurement system.

### Demonstration of the FICS effect

In general, the measurements were performed inside a dark box to avoid excitation of charge carriers by environmental light. In the first step, the phosphor samples were pre-heat-treated at 600 K (or 700 K) to remove the charge carriers from traps. Then, the phosphors were gently ground by using an agate rod (weight ~120 g) for different times. The force-induced TL glow curves and TL spectra were recorded by the above LabVIEW-based system.

Stress recording and TL image read-out of the composite films were carried out in a dark room to eliminate the influence of environmental light. Alternatively, the composite film could be covered by a soft light-blocking layer and used in daylight. Different types of stress were applied on the composite films, including load dragging (average force ~ 0.2 N), free falling (impact energy 4.7–5.5 mJ), and rear-ended collision (impact energy ~ 100 mJ). For the TL image read-out, a digital camera (Canon, EOS 5D Mark II) in manual mode and a heating stage preset at 200 °C were used (Fig. 4e).

## Supplementary information

Supplementary Information
