# Supplementary material for: Force-induced charge carrier storage: a new route for stress recording
Source: Light Sci Appl. 2020 Oct 27;9:182. doi: 10.1038/s41377-020-00422-4 (PMC7588465; doi:10.1038/s41377-020-00422-4)
Supplement: Supplementary file 1 — Supplementary Information [file 41377_2020_422_MOESM1_ESM.doc]

Supplementary Information

Force-induced charge carrier storage: A new route for stress recording

Yixi Zhuang*^,1,2^, Dong Tu^3^, Changjian Chen^1,2^, Le Wang^4^, Hongwu Zhang^5^, Hao Xue^1^, Conghui Yuan^1,6^, Guorong Chen^1,6^, Caofeng Pan^7^, Lizong Dai^1,6^, and Rong-Jun Xie*^,1,2^

^1^College of Materials, Xiamen University, Simingnan-Road 422, Xiamen 361005, China

^2^Fujian Provincial Key Laboratory of Materials Genome, Xiamen University, Simingnan-Road 422, Xiamen 361005, China

^3^School of Physics and Technology, Wuhan University, Bayi-Road 299, Wuhan 430072, China

^4^College of Optical and Electronic Technology, China Jiliang University, Xueyuan-Street 258, Hangzhou 310018, China

^5^Institute of Urban Environment, Chinese Academy of Sciences, Jimei-Avenue 1799, Xiamen 361021, China

^6^Fujian Provincial Key Laboratory of Fire Retardant Materials, Xiamen University, Simingnan-Road 422, Xiamen 361005, China

^7^Beijing Institute of Nanoenergy and Nanosystems, Chinese Academy of Sciences, Xueyuan-Road 30, Beijing 100083, China

*Correspondence: zhuangyixi@xmu.edu.cn; rjxie@xmu.edu.cn

**Keywords:** stress recording; non-real-time stress sensing; mechanoluminescent materials; rare-earth-doped materials; energy storage.


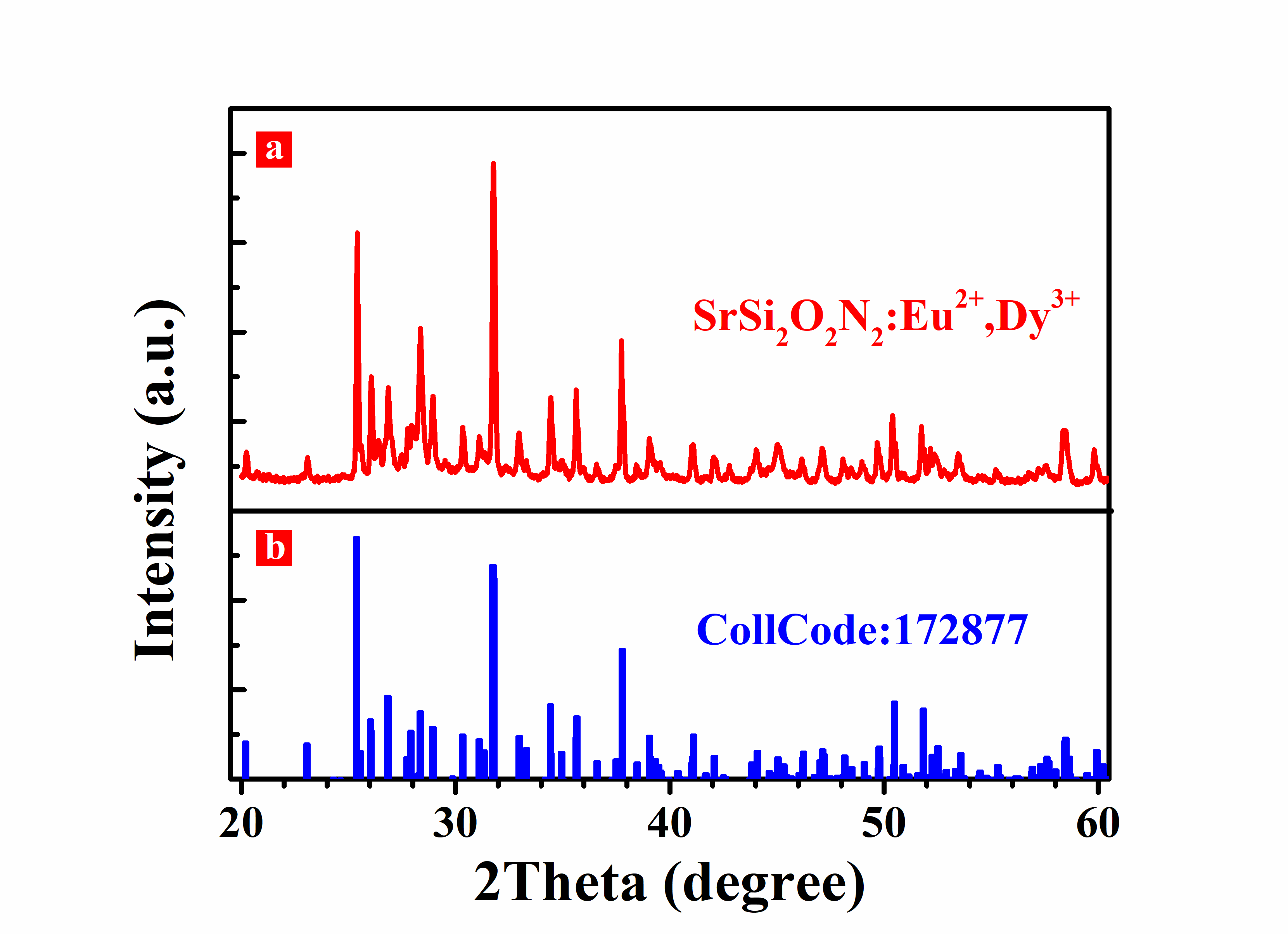


**Fig. S1** **(a)** XRD pattern SrSi_2_O_2_N_2_:Eu^2+^,Dy^3+^. **(b)** The standard diffraction data for SrSi_2_O_2_N_2_ cited from the Inorganic Crystal Structure Database (ICSD), CollCode number: 172877.


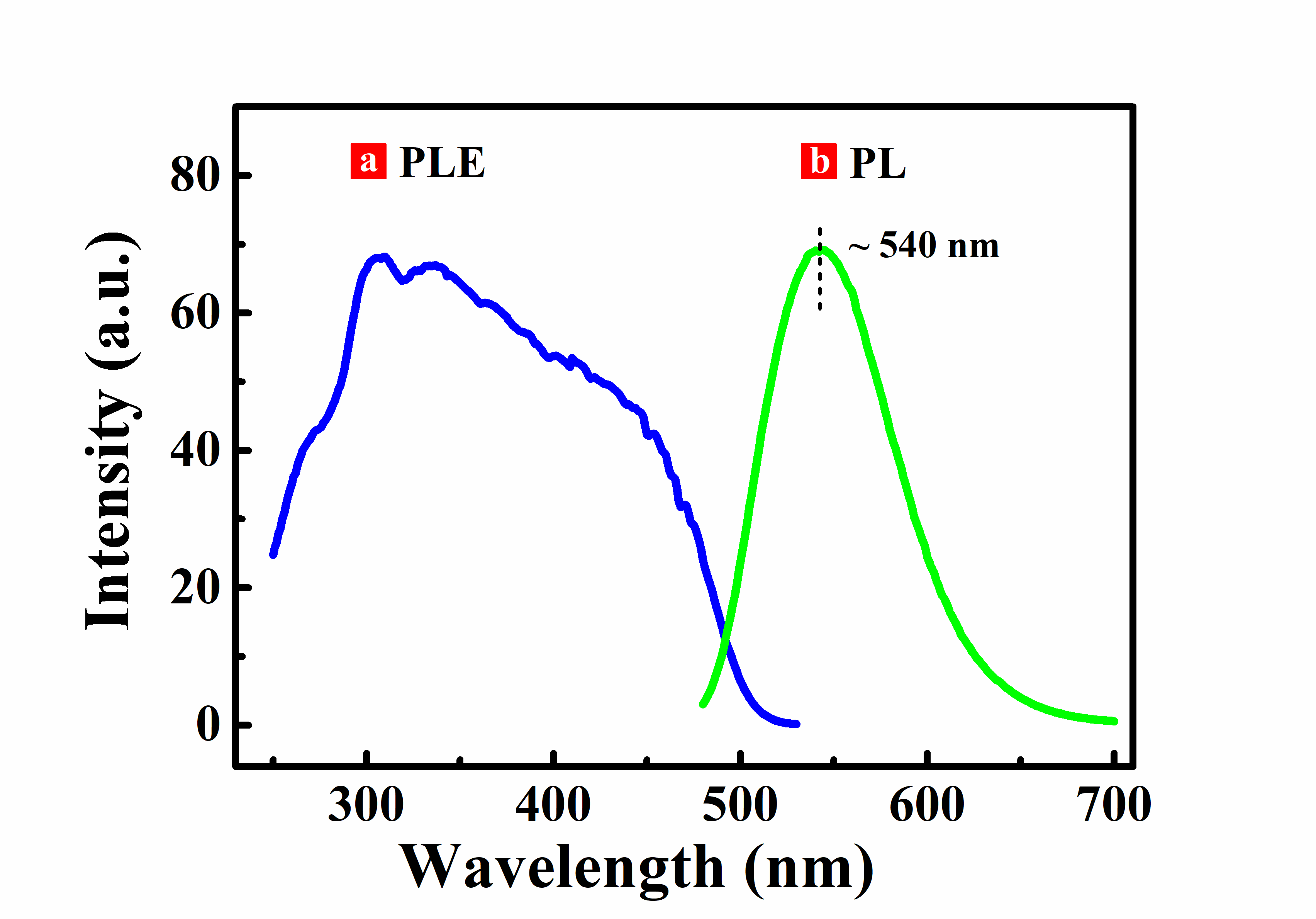


**Fig. S2** **(a)** PLE and **(b)** PL spectra of SrSi_2_O_2_N_2_:Eu^2+^,Dy^3+^. The monitoring and excitation wavelengths for the PLE and PL spectra were 540 and 460 nm, respectively.


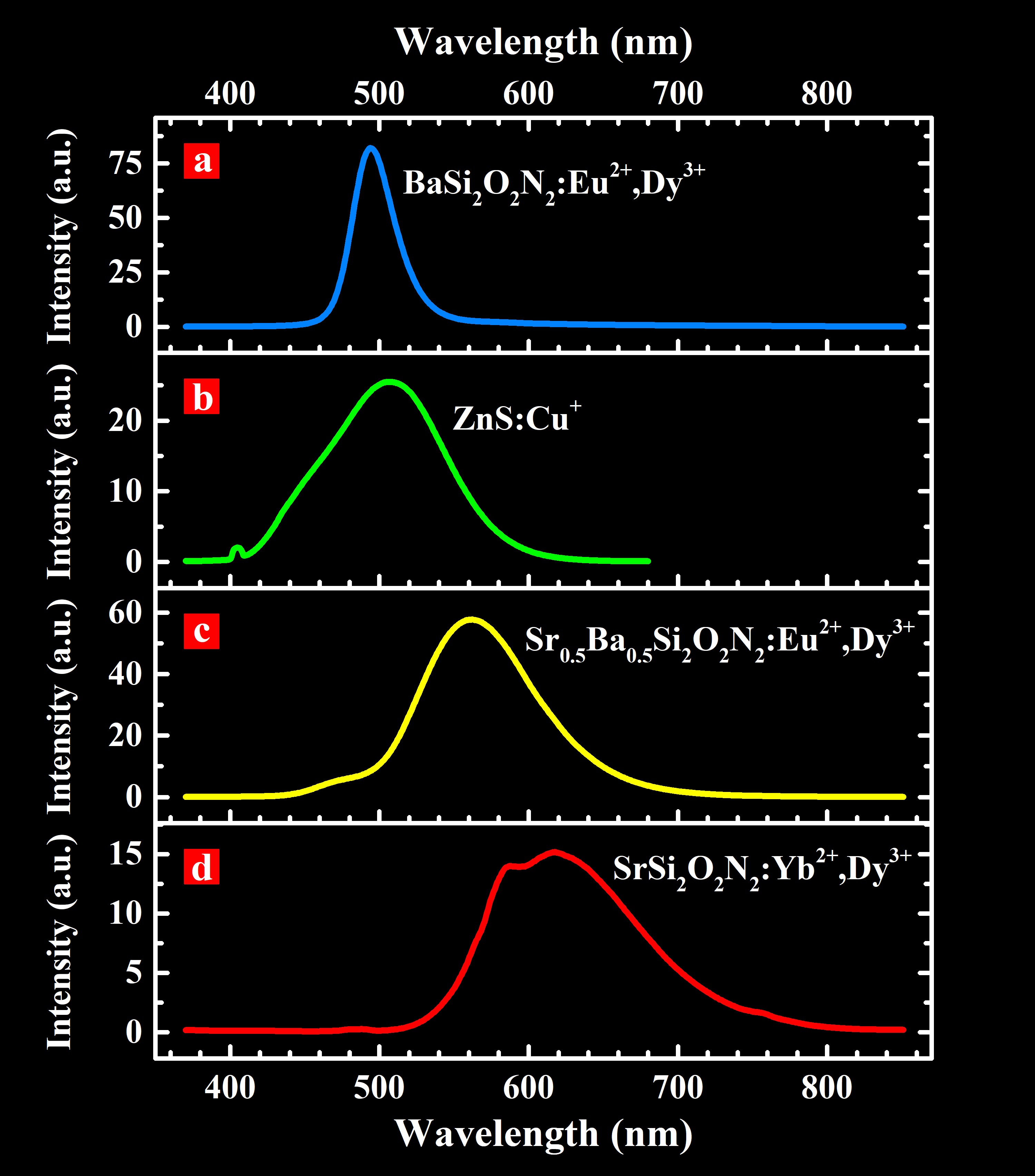


**Fig. S3** PL spectra of the **(a)** BaSi_2_O_2_N_2_:Eu^2+^,Dy^3+^, **(b)** ZnS:Cu, **(c)** (Sr_0.5_Ba_0.5_)Si_2_O_2_N_2_:Eu^2+^,Dy^3+^, and **(d)** SrSi_2_O_2_N_2_:Yb^2+^,Dy^3+^ phosphors at RT. The excitation source was a UV-LED chip with a peak at 365 nm.


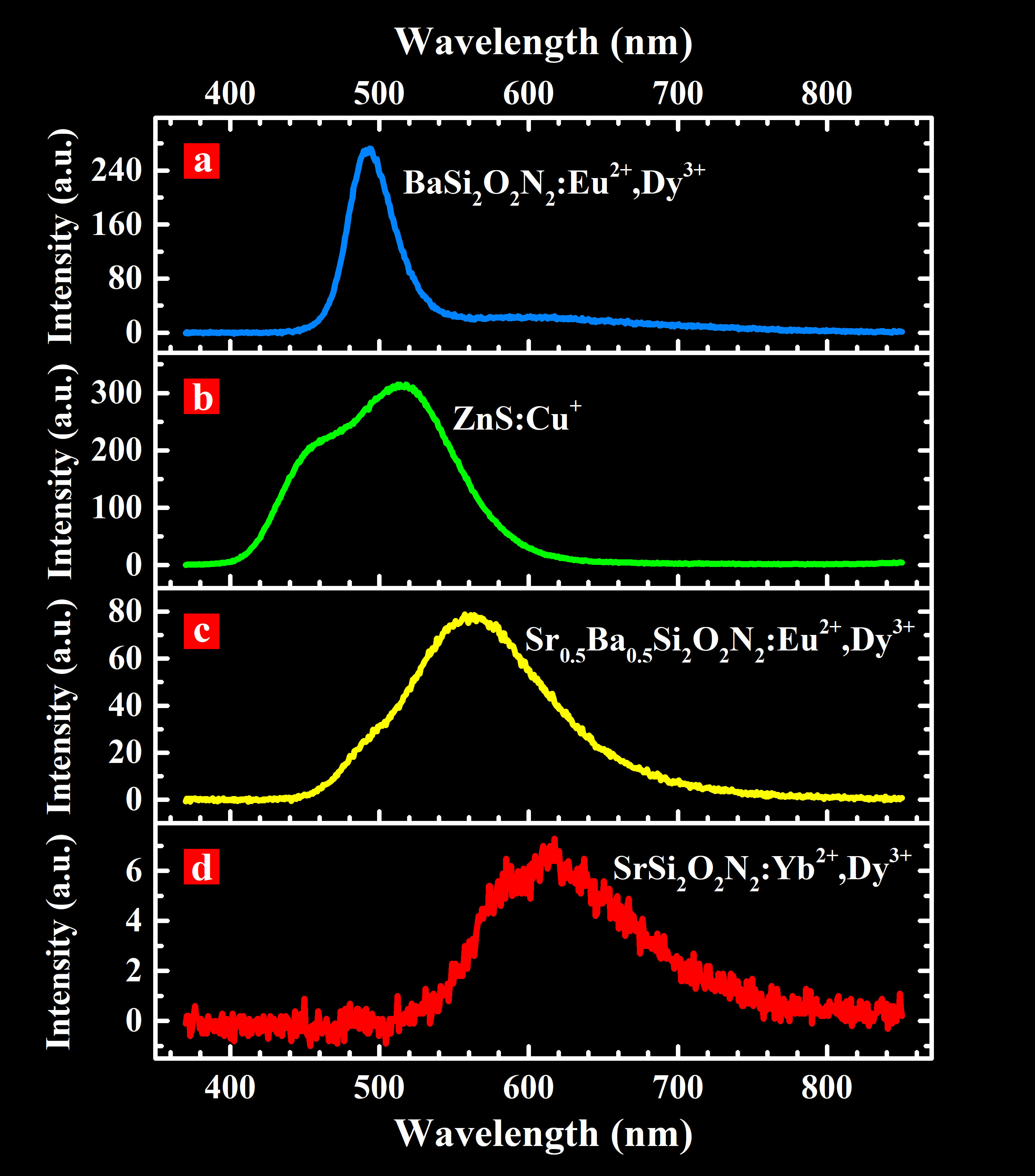


**Fig. S4** ML spectra of the **(a)** BaSi_2_O_2_N_2_:Eu^2+^,Dy^3+^, **(b)** ZnS:Cu, **(c)** (Sr_0.5_Ba_0.5_)Si_2_O_2_N_2_:Eu^2+^,Dy^3+^, and **(d)** SrSi_2_O_2_N_2_:Yb^2+^,Dy^3+^ phosphors under grinding at RT. For each sample, the ML spectra shown in this figure was obtained by averaging 10 spectra. All the samples were thermally bleached at 627 K for 1 min before the ML measurement.


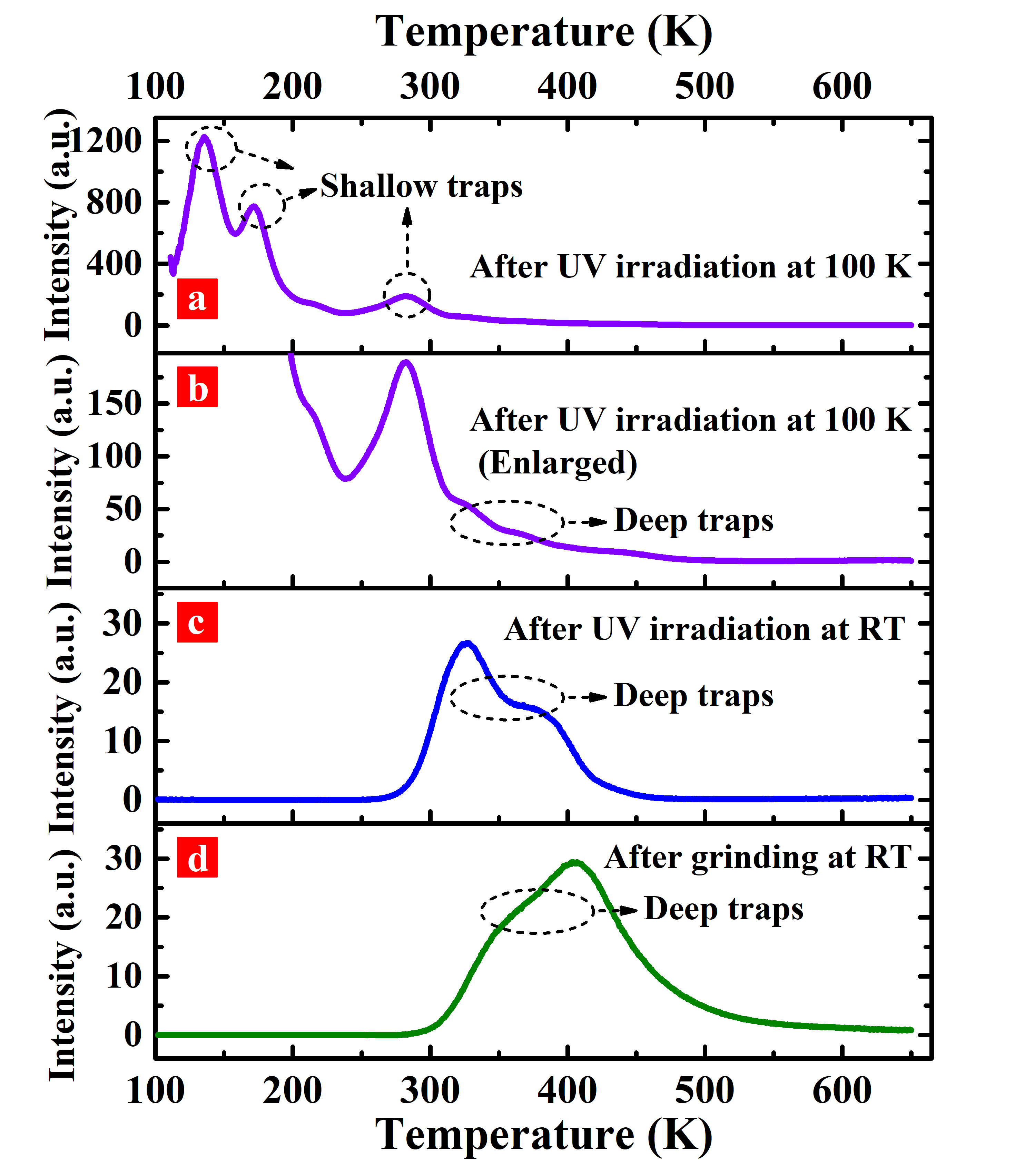


**Fig. S5** TL glow curves of the BaSi_2_O_2_N_2_:Eu^2+^,Dy^3+^ phosphor **(a-b)** after UV irradiation at 100 K, **(c)** after UV irradiation at RT, or **(d)** after grinding at RT. **(b)** is the enlargement of **(a)**. The BaSi_2_O_2_N_2_:Eu^2+^,Dy^3+^ phosphor contained a large number of shallow traps (TL glow peak lower than RT) and also deep traps (higher than RT).


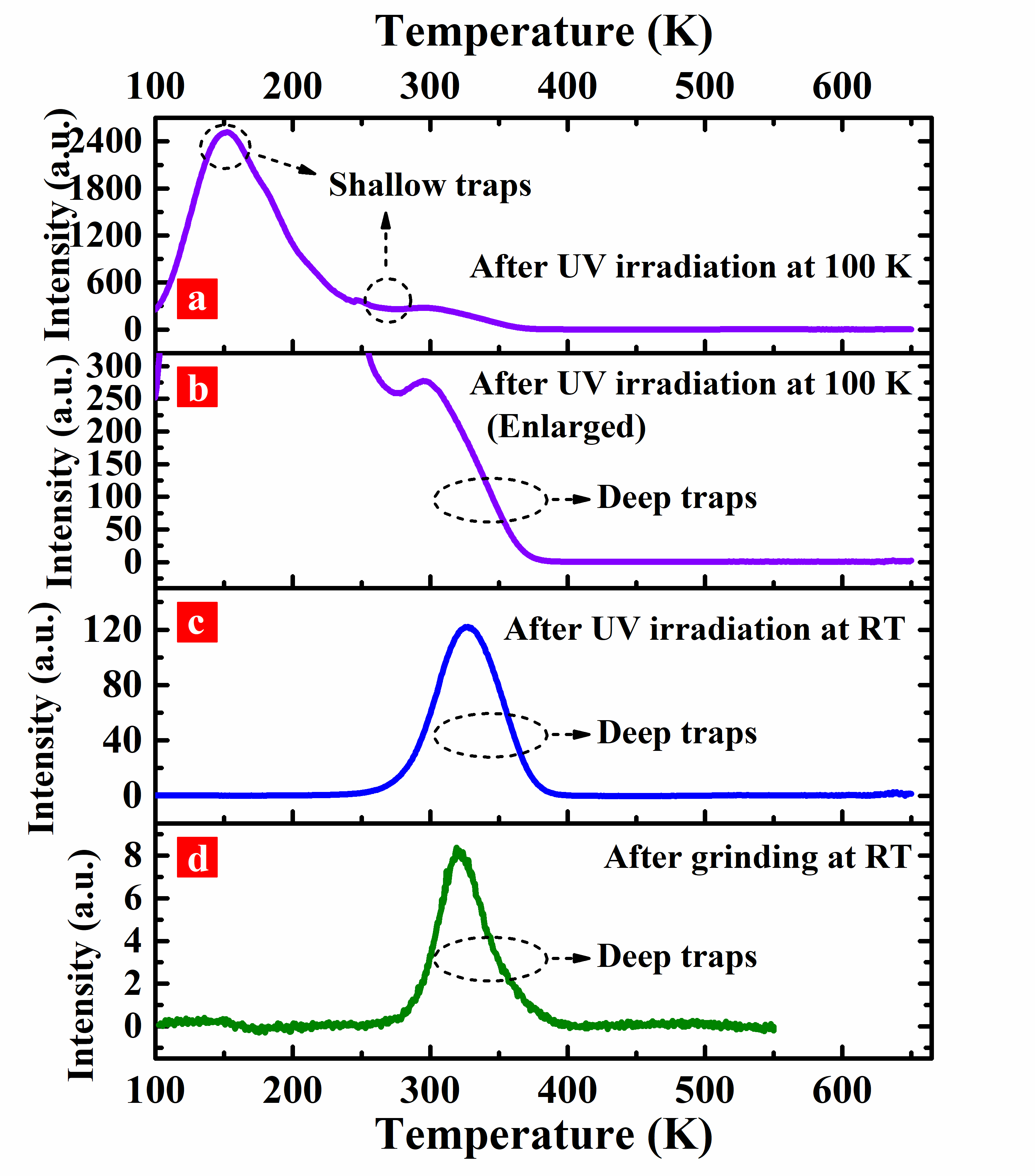


**Fig. S6** TL glow curves of the ZnS:Cu phosphor **(a-b)** after UV irradiation at 100 K, **(c)** after UV irradiation at RT, or **(d)** after grinding at RT. **(b)** is the enlargement of **(a)**. The ZnS:Cu phosphor contained a large number of shallow traps (TL glow peak lower than RT) and also deep traps (higher than RT).


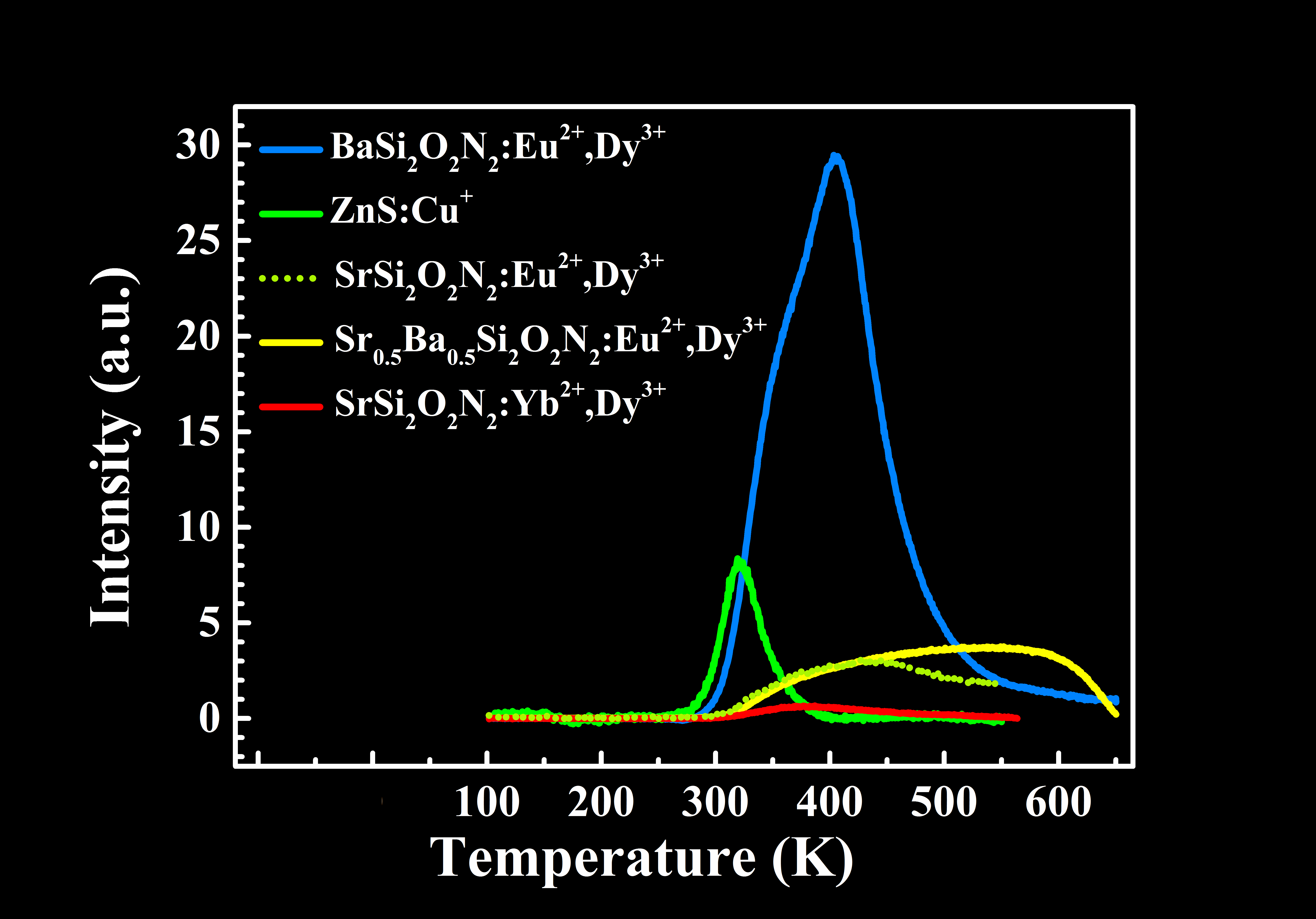


**Fig. S7** Force-induced TL glow curves of various phosphors. Before the measurement, the phosphors were treated by thermal bleaching (600 or 700 K) and grinded for 200 s in the dark.


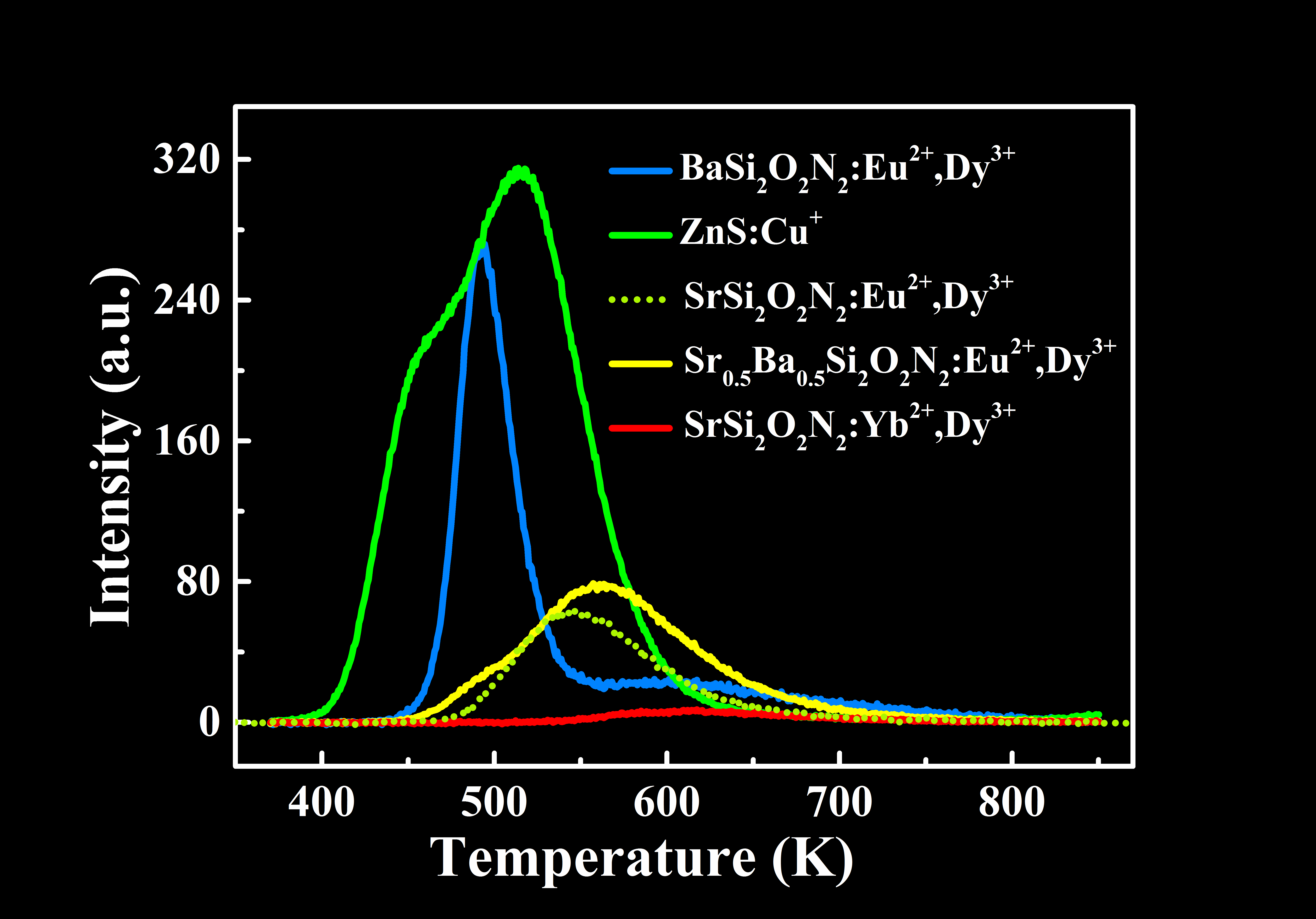


**Fig. S8** ML spectra of various phosphors under grinding at RT. For each sample, the ML spectra shown in this figure was obtained by averaging 10 spectra. This figure indicated that the BaSi_2_O_2_N_2_:Eu^2+^,Dy^3+^ and ZnS:Cu phosphors presented stronger ML than others.


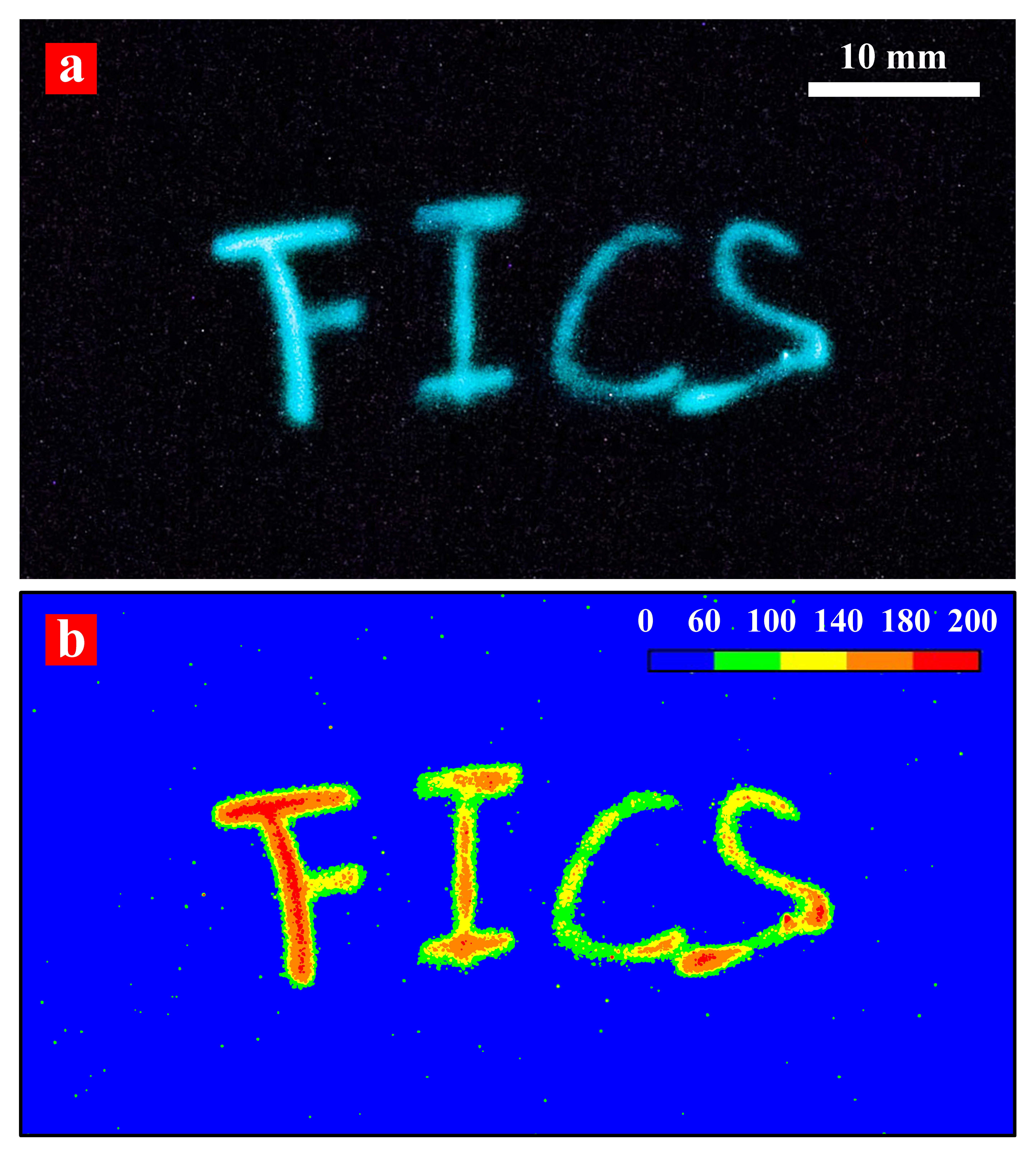


**Fig. S9** **(a)** TL image and **(b)** TL intensity map (pseudo-colour image) read out from the composite film. The film had “F I C S” written on it by using a pen. This result is for the **second** cycle of stress recording and read out on this film.


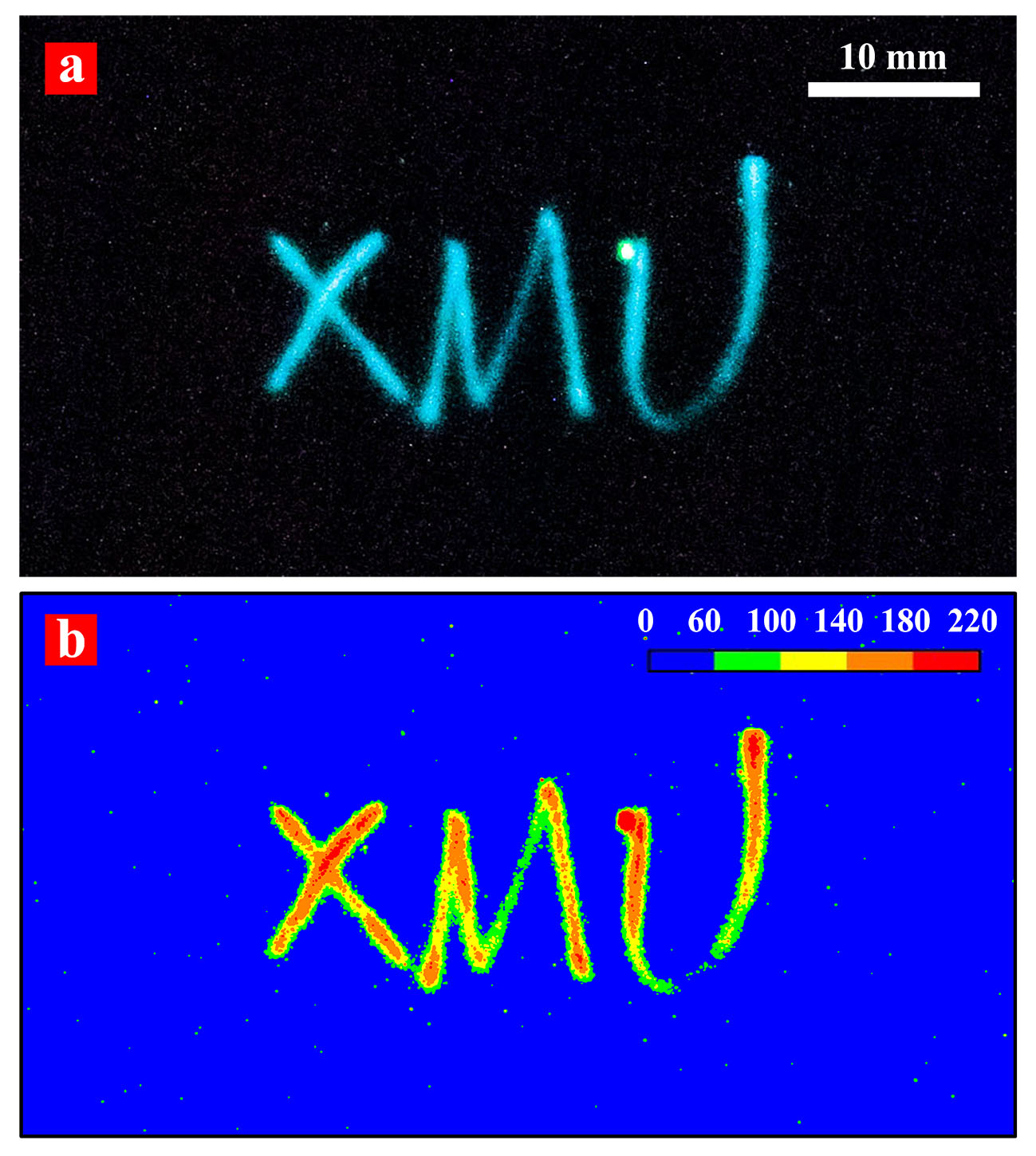


**Fig. S10.** **(a)** TL image and **(b)** TL intensity map (pseudo-colour image) read out from the composite film. The film had “X M U” written on it by using a pen. This result is for the **third** cycle of stress recording and read out on this film.


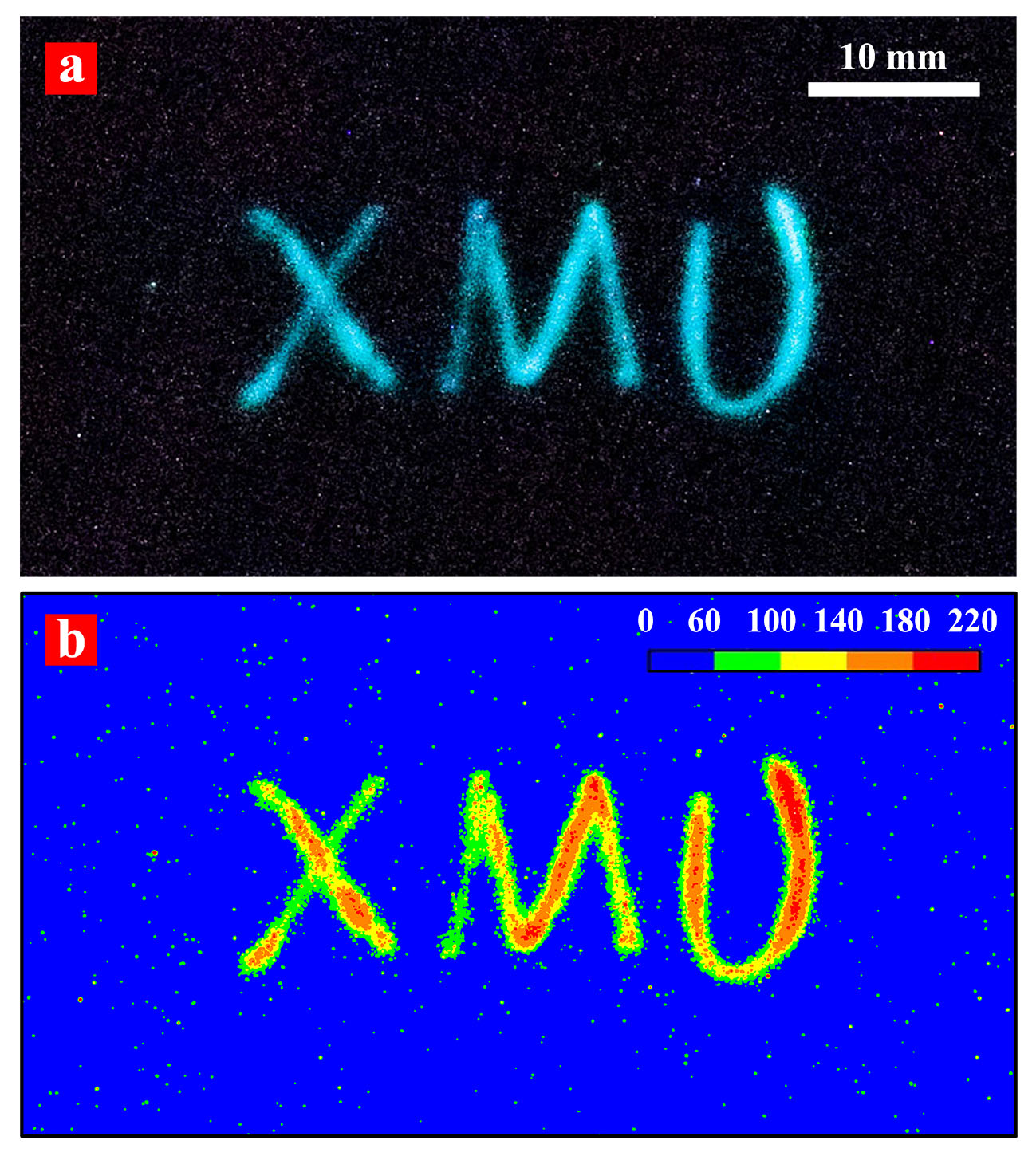


**Fig. S11** **(a)** TL image and **(b)** TL intensity map (pseudo-colour image) read out from the composite film. The film had “X M U” written on it by using a pen. This result is for the **fourth** cycle of stress recording and read out on this film.


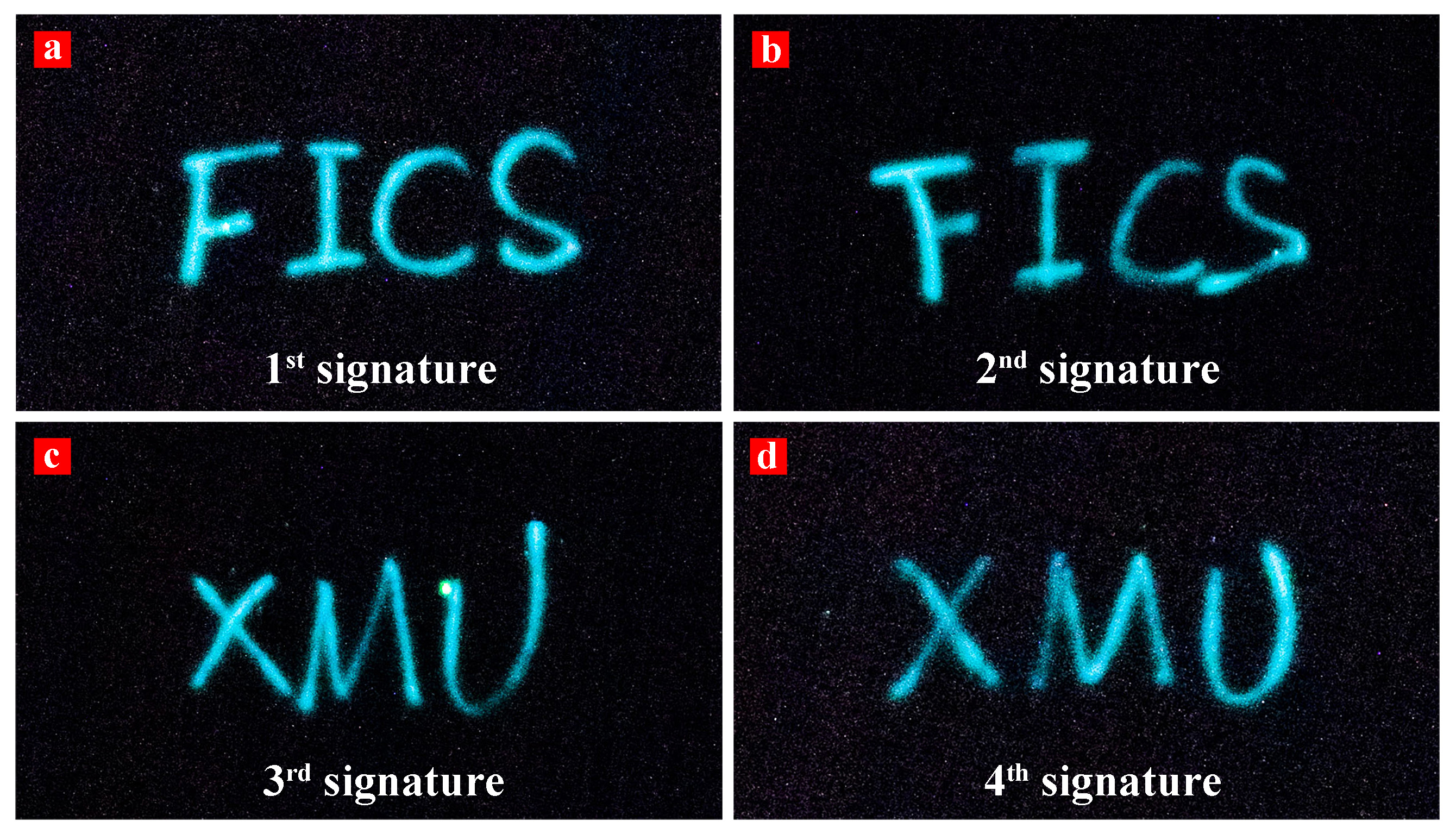


**Fig. S12** Images of force-induced TL read out from the same composite film for four times. After a signature was read out (200 ºC), the signature trace could be removed by heating to a higher temperature (300 ºC). The film was renewed and was able to record new signature.


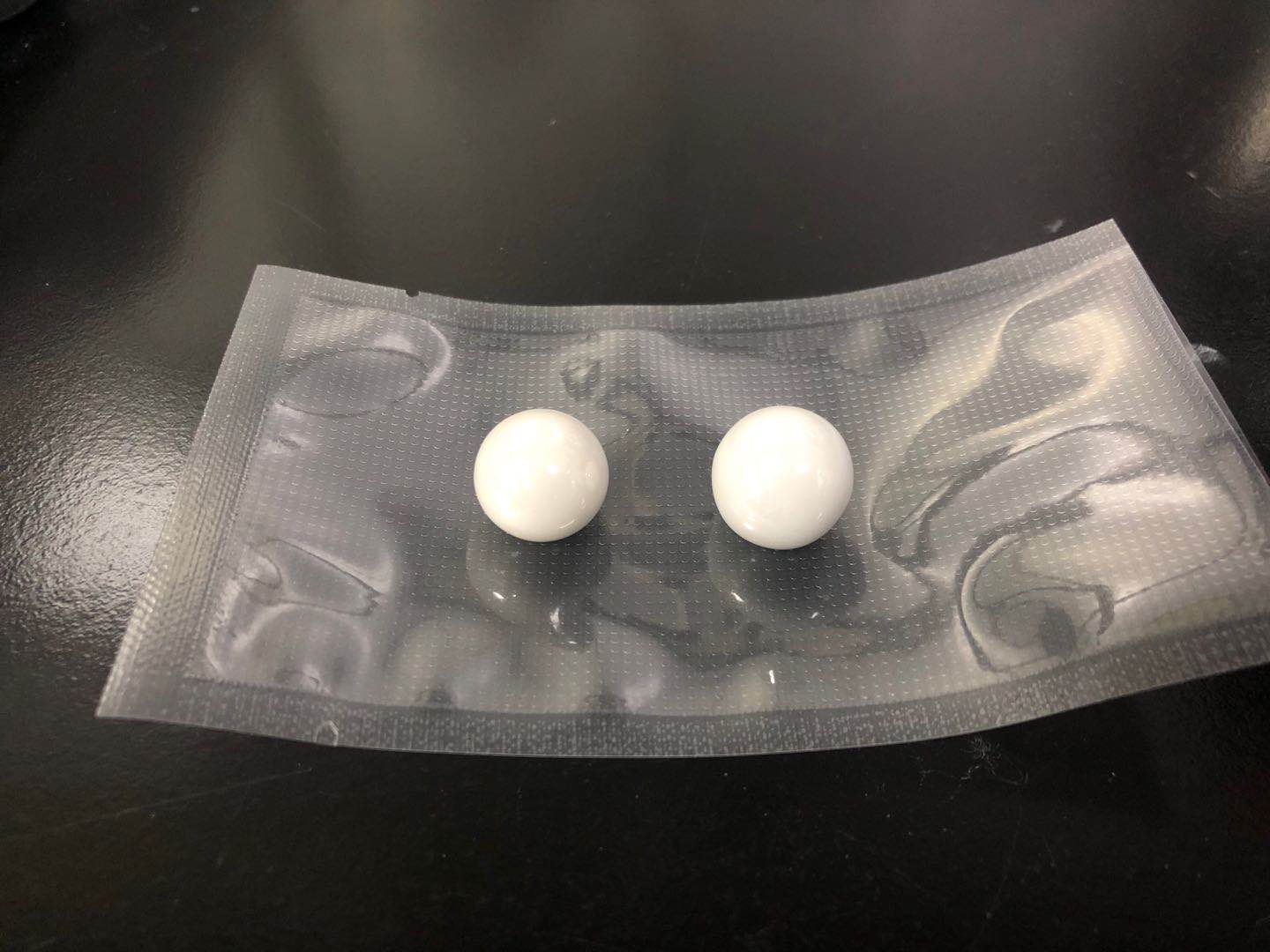


**Fig. S13** White agate balls used for the demonstration of stress recording application (falling point monitoring). The weight of left and right balls are 1.20 and 1.40 g, respectively. The balls were both dropped 40 cm above the BaSi_2_O_2_N_2_:Eu^2+^,Dy^3+^ composite film.


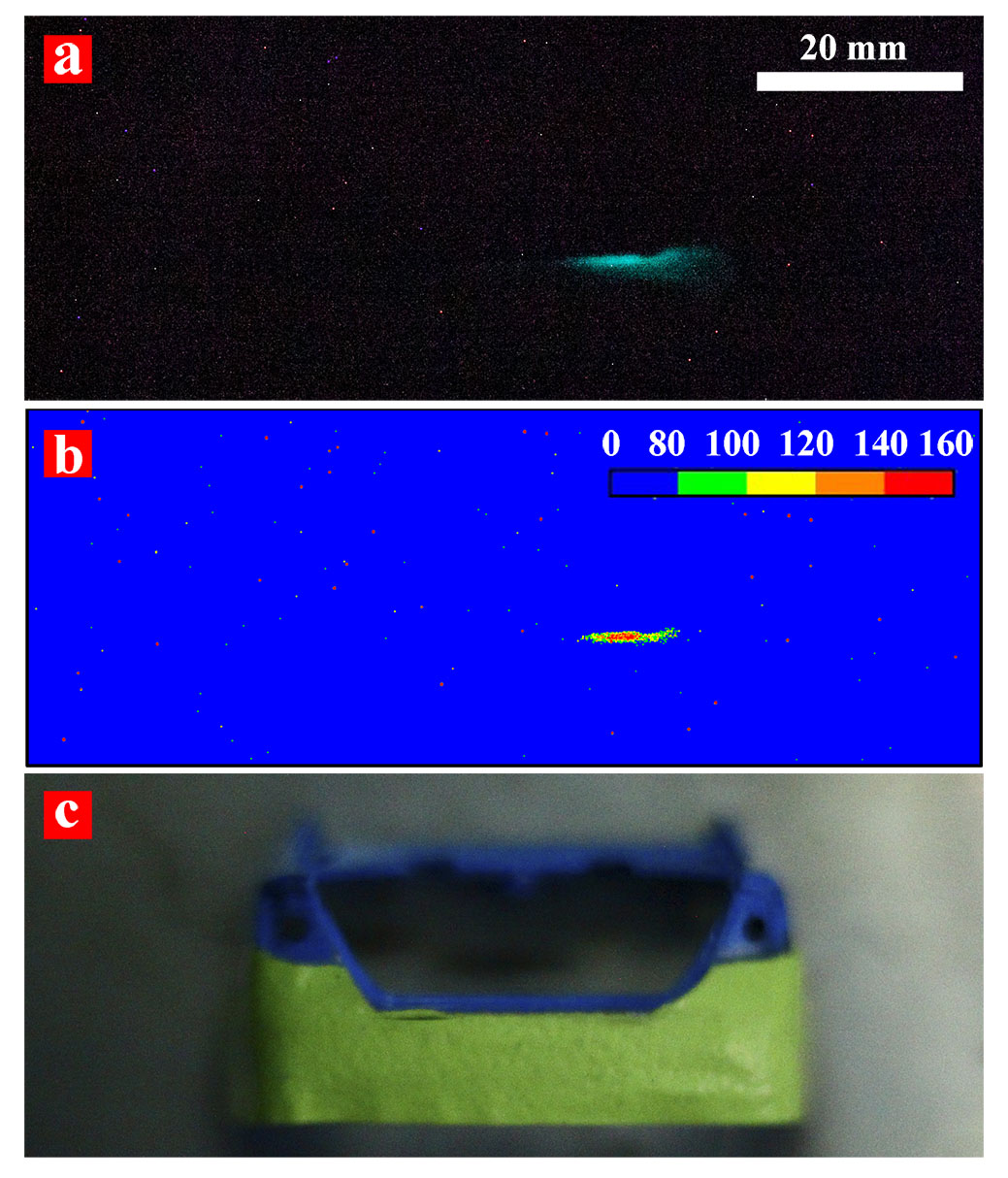


**Fig. S14** **(a)** TL image and **(b)** TL intensity map (pseudo-colour image) read out from the rear of the model car after a collision accident. This result is for the **second** cycle of stress recording and read out. (c) Photograph of the same view field under natural light.


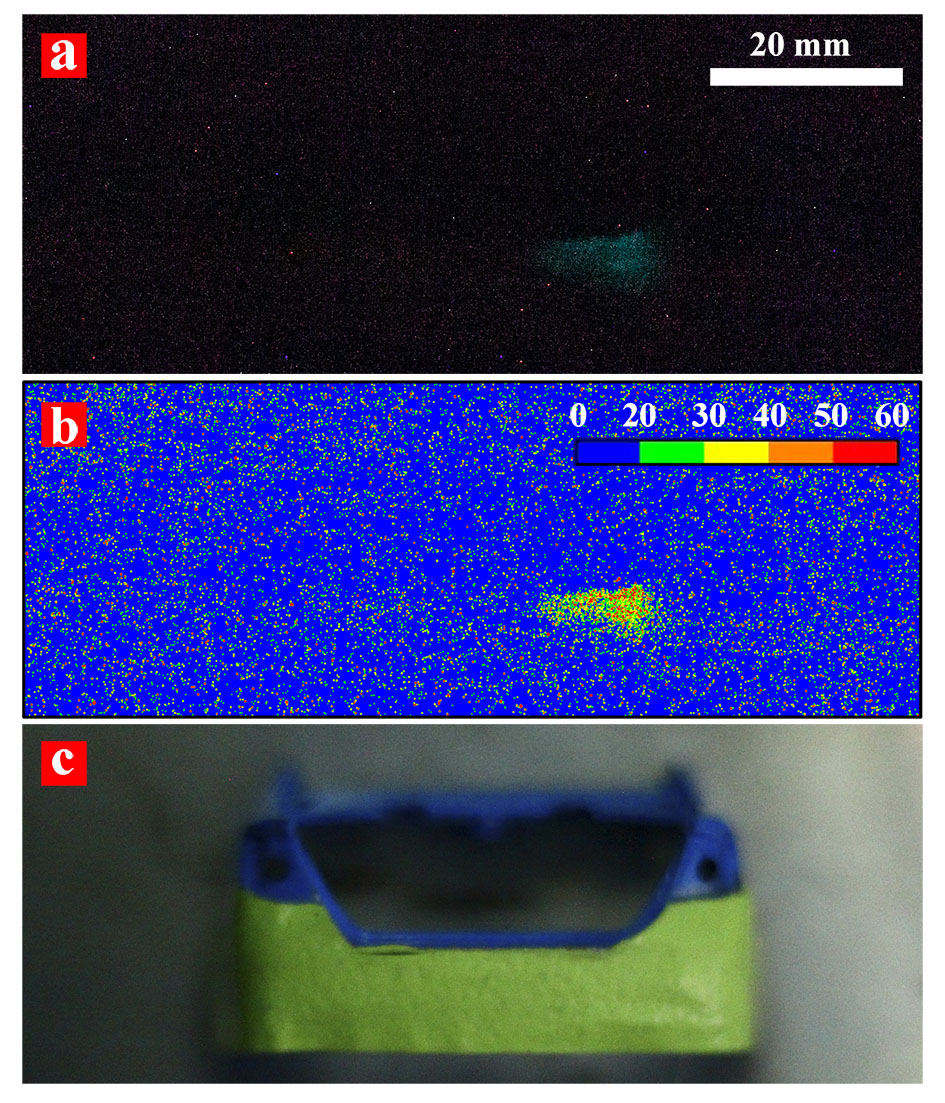


**Fig. S15** **(a)** TL image and **(b)** TL intensity map (pseudo-colour image) read out from the rear of the model car after a collision accident. This result is for the **third** cycle of stress recording and read out. **(c)** Photograph of the same view field under natural light.


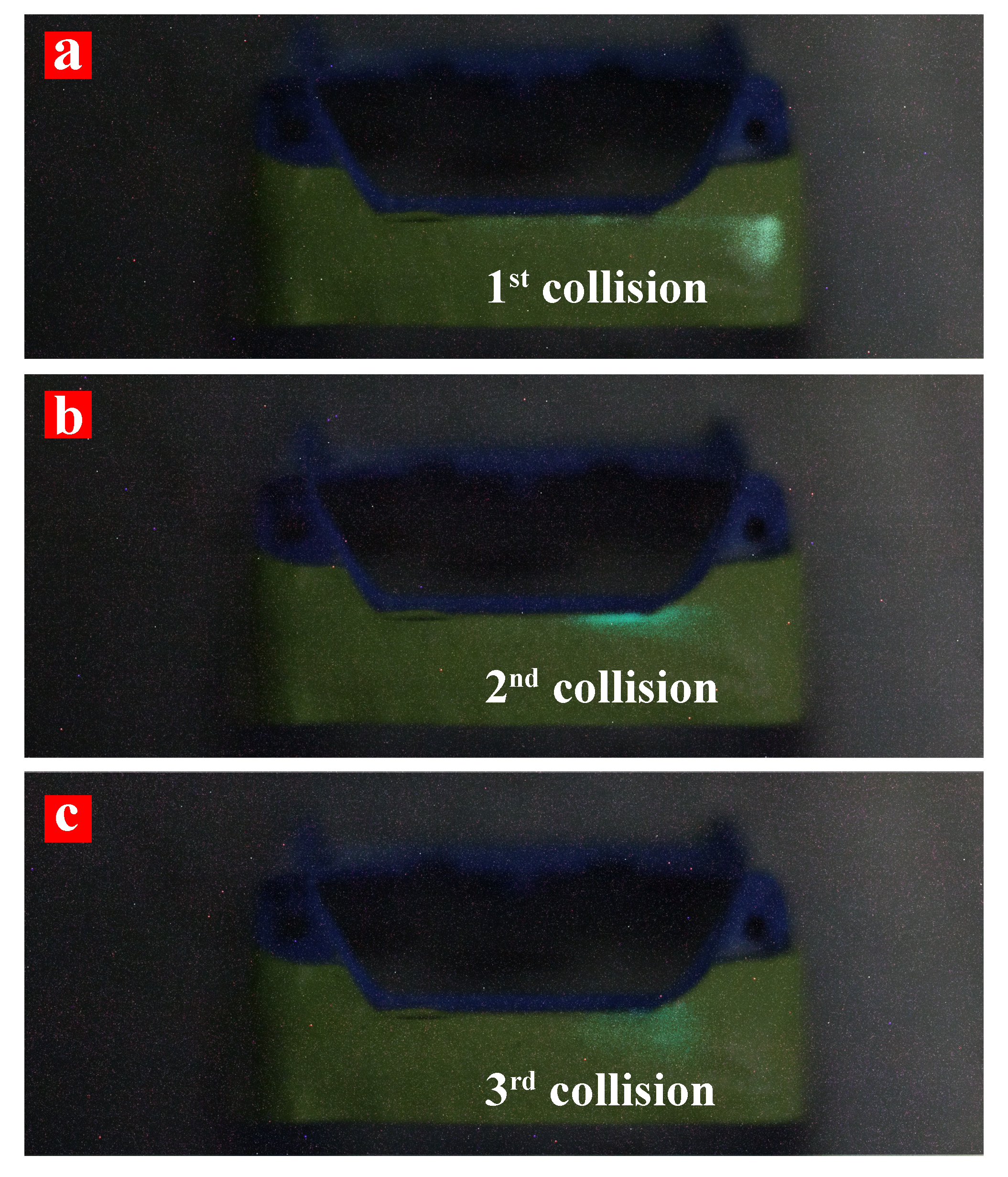


**Fig. S16.** **(a-c)** TL images for three collisions. The TL images were readout by a digital camera when the rear part was heated to 200 ºC. After the TL was decayed, the rear part was cooled to RT and ready for next collison. These figures were obtained by overlapping the TL images (60% transparency) with daylight images.


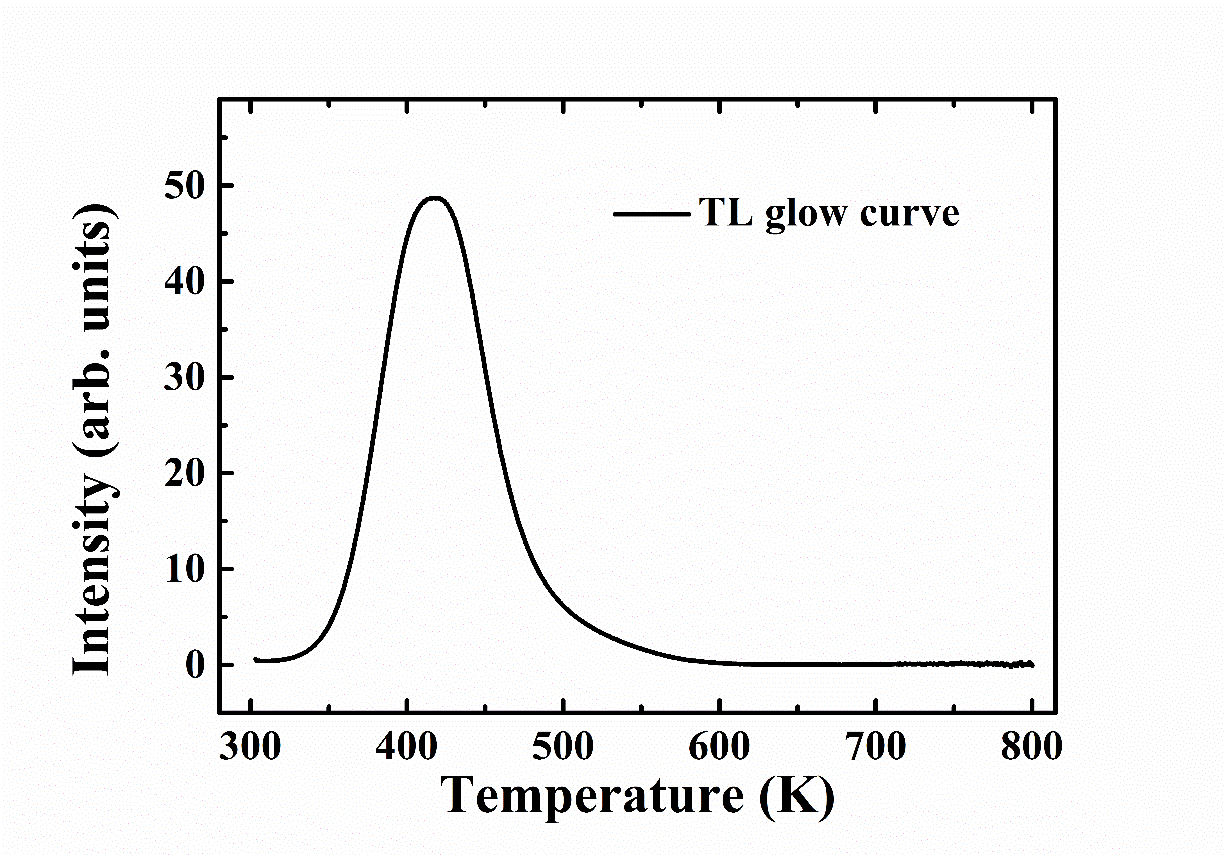


**Fig. S17.** TL glow curve of SrSi_2_O_2_N_2_:Eu^2+^,Dy^3+^ after UV light excitation at RT. Recording range: 300-800 K; heating rate 20 K min^-1^.


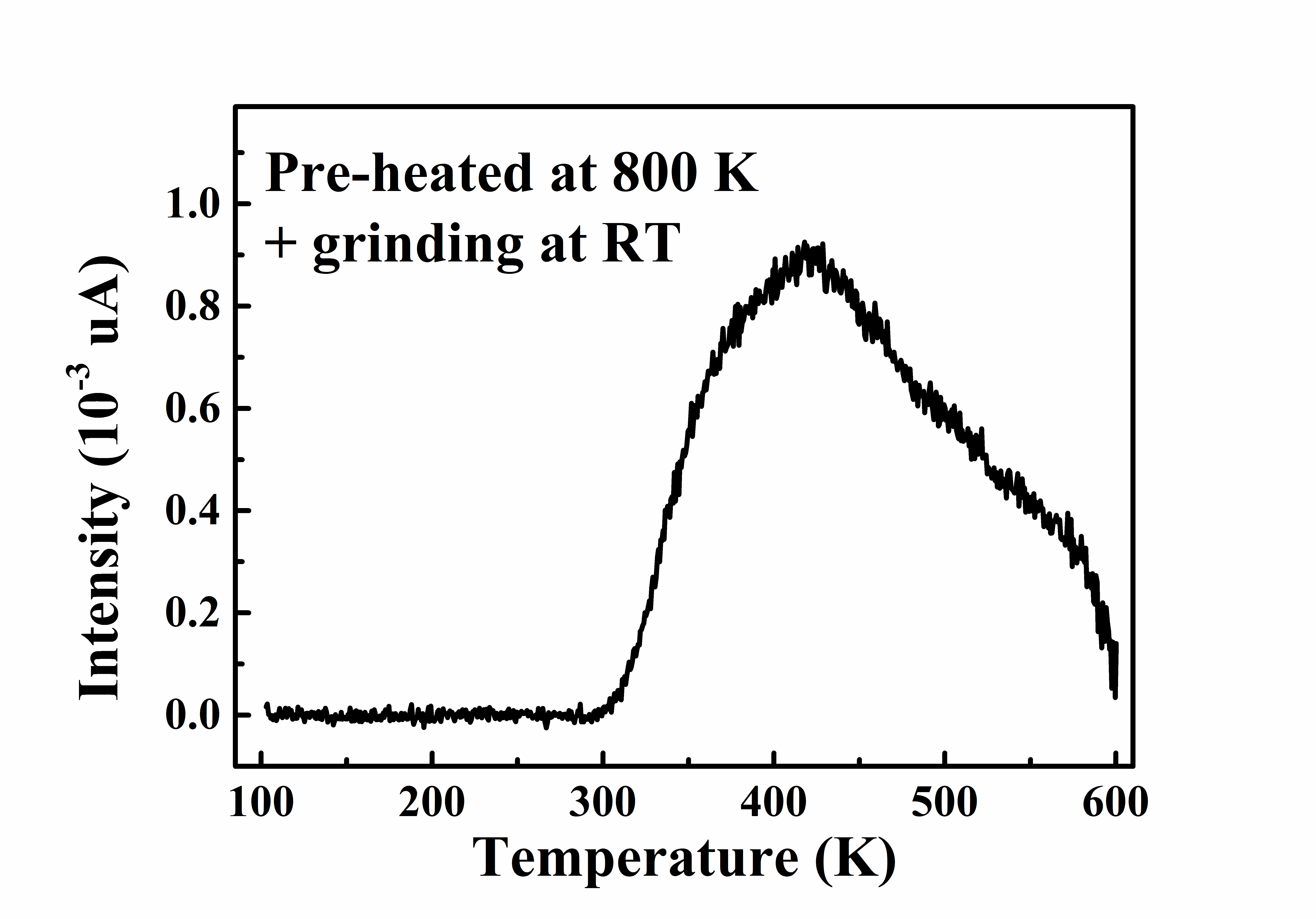


**Fig. S18.** TL glow curve of SrSi_2_O_2_N_2_:Eu^2+^,Dy^3+^ after grinding for 300 seconds at RT. Before grinding, the phosphors were pre-heated to 800 K. Recording range: 100-600 K; heating rate 20 K min^-1^.


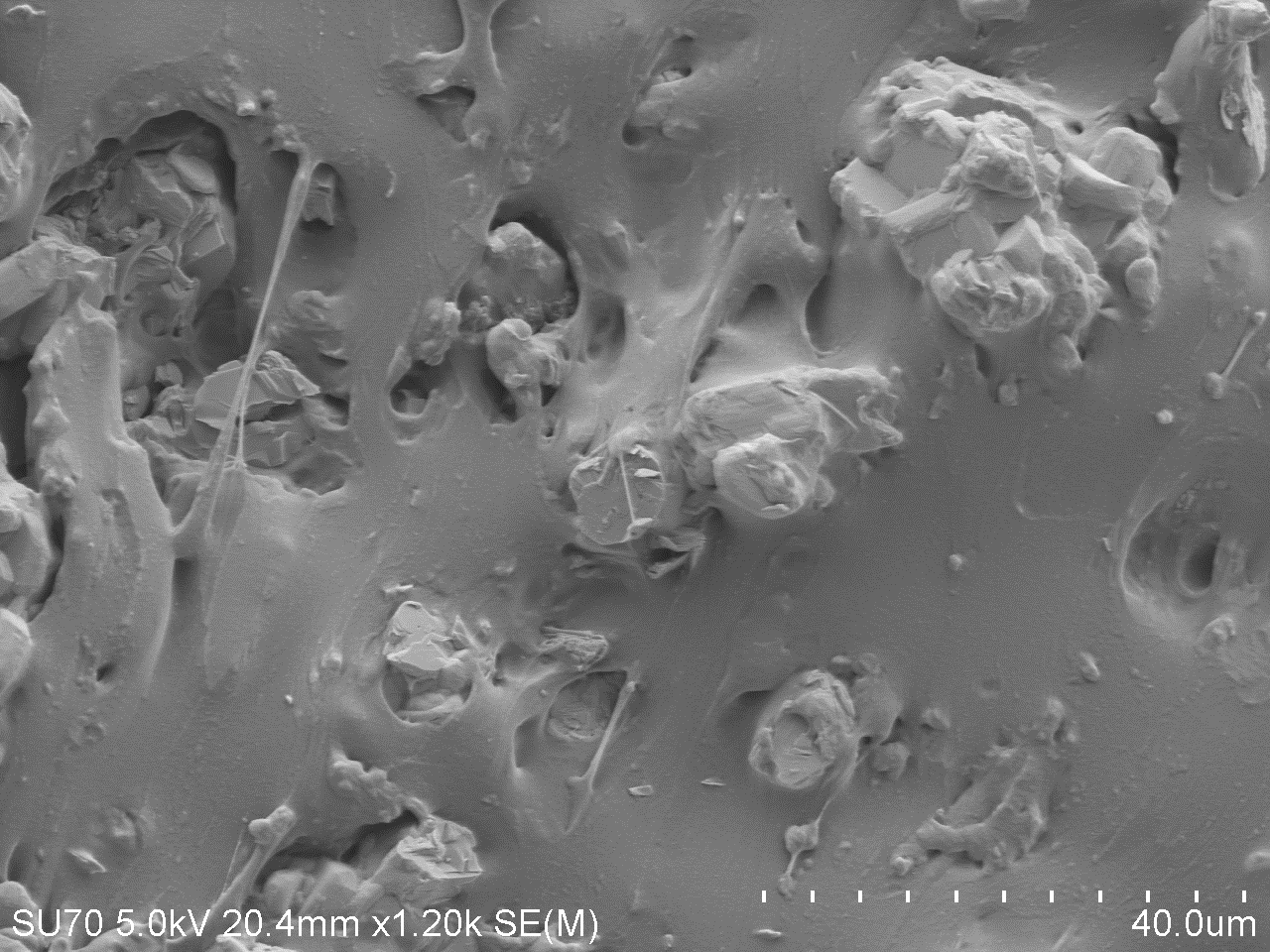


**Fig. S19.** SEM image of the BaSi_2_O_2_N_2_:Eu^2+^,Dy^3+^ composite film after load dragging on the surface. The average load was approximately 0.2 N with a stress area of ~0.8 mm^2^. The comparison of the SEM images before and after load dragging indicated that the particles were hardly fractured by the force, possibly due to the buffering of the soft polymer matrix.
